# Role of gut-derived bacterial lipopolysaccharide and peripheral TLR4 in immobilization stress-induced itch aggravation in a mouse model of atopic dermatitis

**DOI:** 10.1038/s41598-024-56936-z

**Published:** 2024-03-15

**Authors:** Da-Eun Cho, Joon-Pyo Hong, Yoongeun Kim, Ju Yeon Sim, Heenam Stanley Kim, Song-rae Kim, Bombi Lee, Hyo-Sung Cho, Ik-Hyun Cho, Sooan Shin, Mijung Yeom, Soon-Kyeong Kwon, In-Seon Lee, Hijoon Park, Kyuseok Kim, Dae-Hyun Hahm

**Affiliations:** 1 Department of Biomedical Sciences, Graduate School, Kyung Hee University, Seoul, 02447 Republic of Korea; 2 Division of Biosystems and Biomedical Sciences, College of Health Sciences, Korea University, Seoul, 02841 Republic of Korea; 3 Chuncheon Center, Korea Basic Science Institute (KBSI), Chuncheon, 24341 Republic of Korea; 4 Acupuncture and Meridian Science Research Center, Kyung Hee University, Seoul, 02447 Republic of Korea; 5 Department of Korean Medical Science, Graduate School, Kyung Hee University, Seoul, 02447 Republic of Korea; 6 ACCURIEBIO Co., IRIS Lab., 6th Floor, Sangwon 12-gil 34, Seongdong-gu, Seoul, 04790 Republic of Korea; 7 Division of Applied Life Science (Brain Korea 21 PLUS), Gyeongsang National University, Jinju, 52828 Republic of Korea; 8 Department of Ophthalmology, Otorhinolaryngology and Dermatology of Korean Medicine, College of Korean Medicine, Kyung Hee University, Seoul, 02447 Republic of Korea; 9 Department of Physiology, College of Medicine, Kyung Hee University, Seoul, 02447 Republic of Korea

**Keywords:** Atopic dermatitis, Stress, TLR4, Lipopolysaccharide, Itch, Gut microbiota, Immobilization, Microbiology, Physiology, Diseases, Gastroenterology, Medical research

## Abstract

Psychological stress and intestinal leakage are key factors in atopic dermatitis (AD) recurrence and exacerbation. Here, we demonstrate the mechanism underlying bacterial translocation across intestinal epithelial barrier damaged due to stress and further aggravation of trimellitic anhydride (TMA)–induced itch, which remain unclear, in AD mice. Immobilization (IMO) stress exacerbated scratching bouts and colon histological damage, and increased serum corticosterone and lipopolysaccharide (LPS). Orally administered fluorescein isothiocyanate (FITC)-dextran and surgically injected (into the colon) Cy5.5-conjugated LPS were detected in the serum and skin after IMO stress, respectively. The relative abundance of aerobic or facultative anaerobic bacteria was increased in the colon mucus layer, and *Lactobacillus murinus*, *E. coli*, *Staphylococcus nepalensis,* and several strains of *Bacillus* sp. were isolated from the spleens and mesenteric lymph nodes. Oral antibiotics or intestinal permeability blockers, such as lubiprostone (Lu), 2,4,6-triaminopyrimidine (TAP) and ML-7, inhibited IMO stress-associated itch; however, it was reinduced through intradermal or *i.p.* injection of LPS without IMO stress. *I.p.* injection of TAK-242 (resatorvid), a TLR4 inhibitor, abrogated IMO stress-associated itch, which was also confirmed in TLR4-KO mice. IMO stress alone did not cause itch in naïve mice. IMO stress-induced itch aggravation in TMA-treated AD mice might be attributed to the translocation of gut-derived bacterial cells and LPS, which activates peripheral TLR4 signaling.

## Introduction

In recent years, atopic dermatitis (AD) in adults has gained increasing attention because it seriously hinders work life and social activities, leading to poor quality of life. AD symptoms often recur or worsen in adults with a history of AD. Mental stress arising from experiencing disaster and competitive social life is considered the main trigger of the disease^1,2^. Although most dermatologists recognize that stress is related to an imbalance of intestinal immune functions and neuroendocrine circuits governing the stress response that can influence AD progression, the mechanism by which emotional stress aggravates itch sensation and inflammation in the skin of patients with AD has not been elucidated^3,4^.

Chronic stress is also a major cause of irritable bowel syndrome (IBS), the severity of which is related to intestinal epithelial permeability, especially in patients with diarrhea-type IBS^5^. In addition to visceral pain and alterations in bowel habits, patients with IBS show a high prevalence of comorbidities related to allergic skin diseases, such as AD, psoriasis, and rosacea^6,7^. Patients with adult**-**onset AD or a history of AD during childhood have a high risk of developing IBS^8^. These clinical findings suggest that the disruption of intestinal epithelial permeability due to stress may be a considerable factor contributing to the development or aggravation of skin symptoms, such as itch, erythema, and edema, in patients with AD or IBS with skin symptoms. Nevertheless, the mechanisms underlying chronic stress-induced intestinal barrier dysfunction and their role in pruritus aggravation of the skin remain unclear.

Toll-like receptors (TLRs), such as TLR3, 4, and 7, which are presumed to be individually involved in itch sensation, are expressed on small dorsal root ganglia (DRG) neurons^9,10^, as well as keratinocytes and mast cells in the skin^11^. However, activation of TLRs on mast cells, which play a crucial role in regulating Th2-dependent immune responses, does not directly trigger mast cell degranulation that results in allergic reactions, including itch sensation^12,13^. Moreover, direct stimulation of neuronal TLR4 with lipopolysaccharide (LPS) cannot excite itch sensory neurons or induce itch sensation in mouse skin^14^. The roles of several types of TLRs in sensory neurons in chronic itch conditions and the underlying mechanism of their role in itch sensation have not yet been investigated. The present study aimed to investigate the underlying mechanism of itch aggravation in AD skin by psychological stress using trimellitic anhydride (TMA)-induced allergic contact hypersensitivity as a chronic AD model. This study revealed that repeated immobilization (IMO) stress results in the dysbiotic expansion of aerobic and facultative anaerobic bacteria in the intestinal and colonic mucosal layers, some of which and/or their LPS molecules are translocated through the damaged colon epithelial barrier, suggesting their role in exacerbating scratching behavior.

## Methods

### Animals

Ten-week-old BALB/c male mice (Samtaco BIO KOREA, Osan-si, Republic of Korea) and BALB/c nude male mice (Nara**-**Biotec, Seoul, Republic of Korea) weighing 26–28 g and nine-week-old TLR4 KO male mice with a C57BL/6 background weighing 25–27 g (gift from Prof. I-H Cho (Kyung Hee University, Republic of Korea) were housed in a limited-access rodent facility with five mice per polycarbonate cage. The room controls were set to maintain the temperature at 22 ± 2 °C and a relative humidity of 55 ± 15%; the cages were lit by artificial light in a 12-h light/dark cycle and fed food and water ad libitum each day. Animal experiments were performed in accordance with the guidelines for the Care and Use of Laboratory Animals (NIH Publications No. 80-23, revised in 1996) and the ARRIVE guidelines, and approved by the Kyung Hee University Institutional Animal Care and Use Committee (KHSASP-21-089 and KHSASP-22-615).

### Study design

This study included nine independent animal cohorts. Detailed information on the cohort design, experimental procedure, and relevant materials used are provided in the Supplemental Methods and Fig. S1.

### Development of AD-like skin symptoms and IMO stress

**Figure 1:**
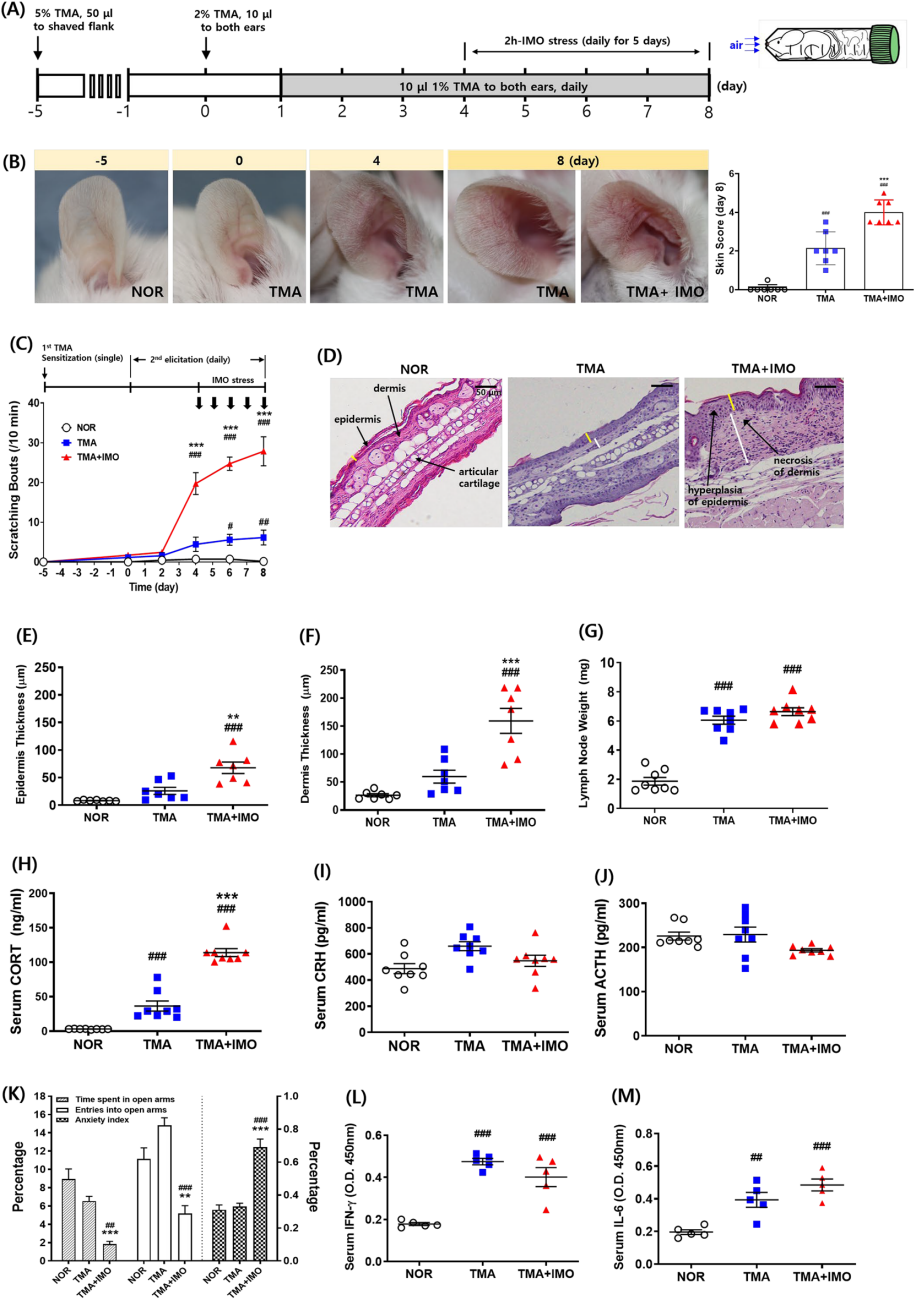
An experimental schedule of inducing AD and application of IMO stress (**A**), pictures of ear and skin, and skin score graph (**B**), and scratching bouts (**C**) in a mouse model of AD. (**D**–**F**) Epidermis (**E**) and dermis (**F**) thickness based on hematoxylin and eosin-stained images (**D**), cervical lymph node weight (**G**), and serum levels of CORT (**H**), CRH (**I**), ACTH (**J**), IFN-γ (**L**), and IL-6 (**M**) were measured. EPM data are presented as relative percentages among groups (**K**). In EPM test, time spent in the open arms and open arm entries/total arm entries were recorded during a 5 min test session (n = 8 mice per group) in cohort 1. The anxiety index was calculated according to *Cohen* et al. as follows^2^: Anxiety Index = 1-[([Open arm time/Test duration] + [Open arms entries/Total number of entries])/2]. At least three images of ear tissue sections per mouse were used to measure the epidermal and dermal thickness in E and F. The time points of IMO stress (a total of 5 instances) are denoted by the black arrows in C. Yellow and white bars indicate the thickness of the epidermis and dermis, respectively, in D. Scale bar = 50 μm in D. Error bars represent SEM ^#^*p* < 0·05, ^##^*p* < 0·01, ^###^*p* < 0·001 versus NOR group; ^**^p < 0·01, ^***^p < 0·001 versus TMA group. Two-way analysis of variance was used for EPM data. AD, atopic dermatitis; IMO, immobilization; CORT, corticosterone; CRH, corticotrophin-releasing hormone; ACTH, adrenocorticotropic hormone; IFN, interferon; IL, interleukin. TMA, trimellitic anhydride; NOR, normal; EPM, elevated plus maze.

AD**-**like chronic skin symptoms were induced in mice by treatment with TMA (Sigma‒Aldrich, St. Louis, MO, USA) according to a previously established protocol^15^. Briefly, mice were sensitized once with 50 μL of 5% TMA on shaved flank skin, and both ears were treated with 10 μL of 1% (or 2%) TMA from days 0 to 8 or 12 (elicitation period). During this period, mice underwent 2 h of IMO stress five times during daylight hours under light conditions from days 4 to 8 using a transparent plastic 50 mL Falcon® tube (Greiner, Frickenhausen, Germany) equipped with a ventilation aperture (Fig. 1A). TMA was dissolved in acetone and isopropyl myristate (4:1 v/v) immediately before application.

### Drug treatments

Mice received each solution of 20 and 100 μg/kg lubiprostone (Lu, difluoropentyl-2-hydroxy-6-oxooctahydrocyclopenta-heptanoic acid; Sigma-Aldrich) dissolved in 100 μL maize oil (Sigma**‒**Aldrich)**,** and 3 mg/kg 2,4,6 triaminopyrimidine (TAP; Sigma-Aldrich) and 1 mg/kg ML-7 hydrochloride (1-(5-iodonaphthalene-1-sulfonyl)-1H-hexahydro-1,4-diazepine hydrochloride; TOCRIS, Bristol, UK) dissolved in 100 μL 0.1 M Tris–HCl (Sigma-Aldrich)^16^. Next, 2 mg/kg TAK-242 (6R-[[(2-chloro-4-fluorophenyl)amino]sulfonyl]-1-cyclohexene-1-carboxylic acid, ethyl ester; Sigma**‒**Aldrich) dissolved in 30 μL dimethyl sulfoxide (DMSO, Sigma-Aldrich) was administered via i.p. injection^17^. LPS (purified from *E. coli* O111:B4 by ion-exchange chromatography, and TLR ligand tested; L3024, Sigma**‒**Aldrich) was dissolved in 50 μL saline for i.p. and 20 μL saline (per ear) for i.d. injection*.* Fig. S1 illustrates the administration sequence and timing of each drug as indicated by the injection numbers and corresponding time points. On the last day, the mice were anesthetized with 2% isoflurane, and blood, feces, and organs were collected (Fig. 1A). The antibiotic cocktail (Abx), which consisted of ampicillin (1 mg/mL), gentamicin (1 mg/mL), neomycin (1 mg/mL), and vancomycin (0·5 mg/mL) from Sigma-Aldrich, was administered daily for three days at a dose of 200 μL/mouse by oral gavage.

### Ear skin manifestation and scratching behavior

The mouse ears were imaged using a digital camera (Canon 20D; Canon Inc., Tokyo, Japan). The skin score for both ears was calculated by summing the individual symptom (scaling/dryness, hemorrhage/excoriation, and edema/redness) scores, graded as 0 (no symptoms), 1 (mild), 2 (moderate), or 3 (severe). To assess scratching behavior, the mice were individually placed in a transparent cylinder (11 cm in diameter), and their behavior was recorded using a video camera for 30 min after 30 min acclimation. One bout of scratching was defined as an episode in which the mouse lifted its hind paw and continuously scratched it for any length of time until the paw returned to the floor^18^. The mouse ear was analyzed using a digital camera (Canon 20D; Canon Inc., Tokyo, Japan). The epidermal and dermal thickness of the ear was measured using hematoxylin and eosin (H&E) staining of ear skin tissue.

### Fluorescein isothiocyanate (FITC)-conjugated dextran assay

The mice were orally administered FITC**-**dextran (M.W. 4000; Sigma**‒**Aldrich) dissolved in 100 μL 1X phosphate**-**buffered saline (PBS) solution at a dose of 400 mg/kg after 4 h of fasting (Fig. S1). The mice were then returned to their cages and starved for another 4 h. The blood concentration of FITC**-**dextran was determined using a fluorospectrophotometer (Gemini EM; Molecular Devices, San Jose, CA, USA), with excitation at 485 nm and emission at 528 nm^19^.

### EPM test

The EPM test was performed in cohort 1 on day 8 after the last exposure to IMO stress. Detailed information is provided in the Supplemental Methods section.

### In vivo fluorescence microscopy

On the last day of the daily IMO stress period for 5 days on day 8, nude mice (n ≥ 3 per group) were anesthetized with inhaled isoflurane. After midline laparotomy, the rectum was isolated between silk ties. Cy5·5-labeled bacterial LPS (0·15 mg/mouse; NANOCS, New York, NY, USA) was directly injected into the proximal colon of the isolated intestine. The abdominal wall was closed with silk sutures. The whole**-**body distribution of Cy5·5-labeled LPS was fluorescently imaged using the VISQUE InVivo Smart LF bioimaging system (VIEWORKS, Daejeon, Republic of Korea) with an excitation wavelength of 630–680 nm and emission wavelength of 690–740 nm. The mean radiant efficiency in the region of interest was processed using Clevue software (VIEWORKS).

### Enzyme-linked immunosorbent assay (ELISA)

Serum concentrations of LPS (MBS700021; MyBioSource, San Diego, CA, USA), CORT (KA0468; Abnova, Neihu District, Taipei City, Taiwan), CRH (MBS265444; MyBioSource), ACTH (MBS2700344; MyBioSource), IFN-γ (551,866; BD Bioscience, Franklin Lakes, NJ, USA), and IL-6 (555,240; BD Bioscience) were measured using ELISA kits according to the manufacturer’s protocols.

### Histology

The formalin-fixed ear and colon tissues were embedded in paraffin, cut into 4 μm-thick sections using a manual rotary microtome (Thermo Shandon Finesse 325; Thermo Fisher Scientific, Waltham, MA, USA), and stained with 1% H&E (Sigma-Aldrich). All stained tissue sections were photographed using a microscope (BX53; Olympus Ltd., Tokyo, Japan).

### Immunofluorescence staining

Immunofluorescence staining of LPS molecules, occludin, TLR4, and PGP9.5 was performed on paraffin-embedded sections of colon or ear tissue harvested on day 8. The detailed method is described in the supplemental methods section.

### PCR-based quantitation of total gut bacteria in feces

Genomic DNA was extracted from fecal samples using an SPINeasy DNA kit (MP Biomedicals, Irvine, CA, USA). The primer sequences and operating conditions used for PCR amplification of total bacteria were as follows: forward; 5′-TGGCTCAGGACGAACGCTGGCGGC-3′, reverse; 5′-CCTACTGCTGCCTCCCGTAGGAGT-3′; annealing temperature: 66 °C, 348 bp, and 30 cycles^20^.

### 16S rRNA sequence-based microbiota analysis

Changes in the gut bacterial composition in the colonic mucus layers of naïve and IMO-stressed mice (n = 3) were analyzed by sequencing the bacterial 16S rRNA gene. Detailed information is provided in the Supplemental Methods section.

### Cultural analysis

Bacterial cells translocated through the intestinal epithelial barrier were isolated via anaerobic culture of homogenates of lymphoid organs, such as the spleen and mesenteric lymph nodes, and identified by colony PCR of 16S rRNA genes and DNA sequencing. The detailed method is described in the supplemental methods section.

### Statistical analysis

**Figure 2:**
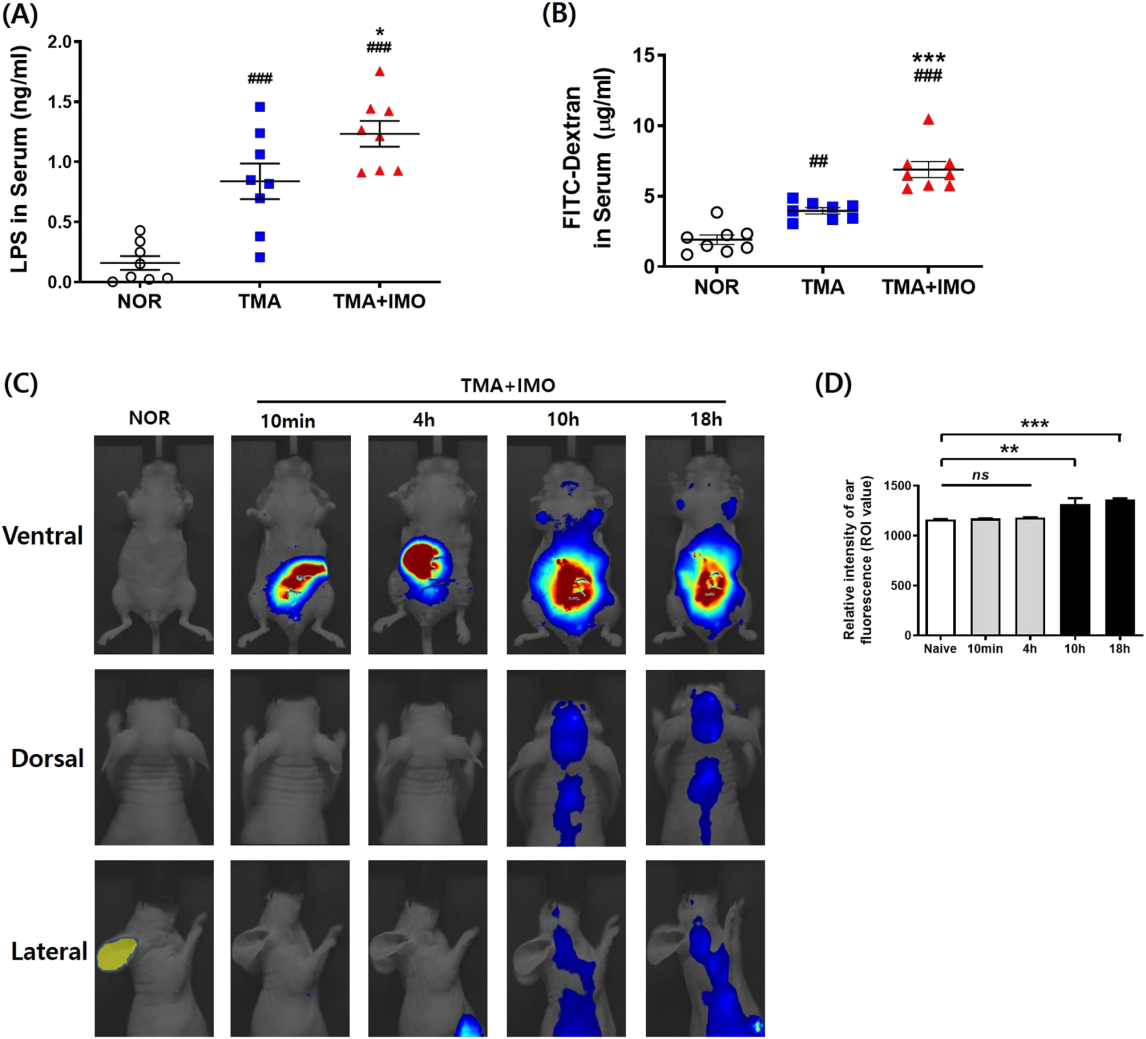
Serum concentrations of LPS molecules (**A**) and FITC-dextran (**B**) after IMO stress in a mouse model of AD. In vivo fluorescence images (**C**) and a bar graph (**D**) showing the time-dependent distribution of Cy5.5-labeled bacterial LPS surgically injected into the colon after IMO stress in TMA-treated mice. Nude male mice on a BALB/c background were used for the in vivo immunofluorescence study. ^##^*p* < 0·01, ^###^*p* < 0·001 versus NOR group; ^*^p < 0·05, ^***^p < 0·001 versus TMA group. LPS, lipopolysaccharides; FITC, fluorescein isothiocyanate; AD, atopic dermatitis; IMO, immobilization; TMA, trimellitic anhydride; NOR, normal.

**Figure 3:**
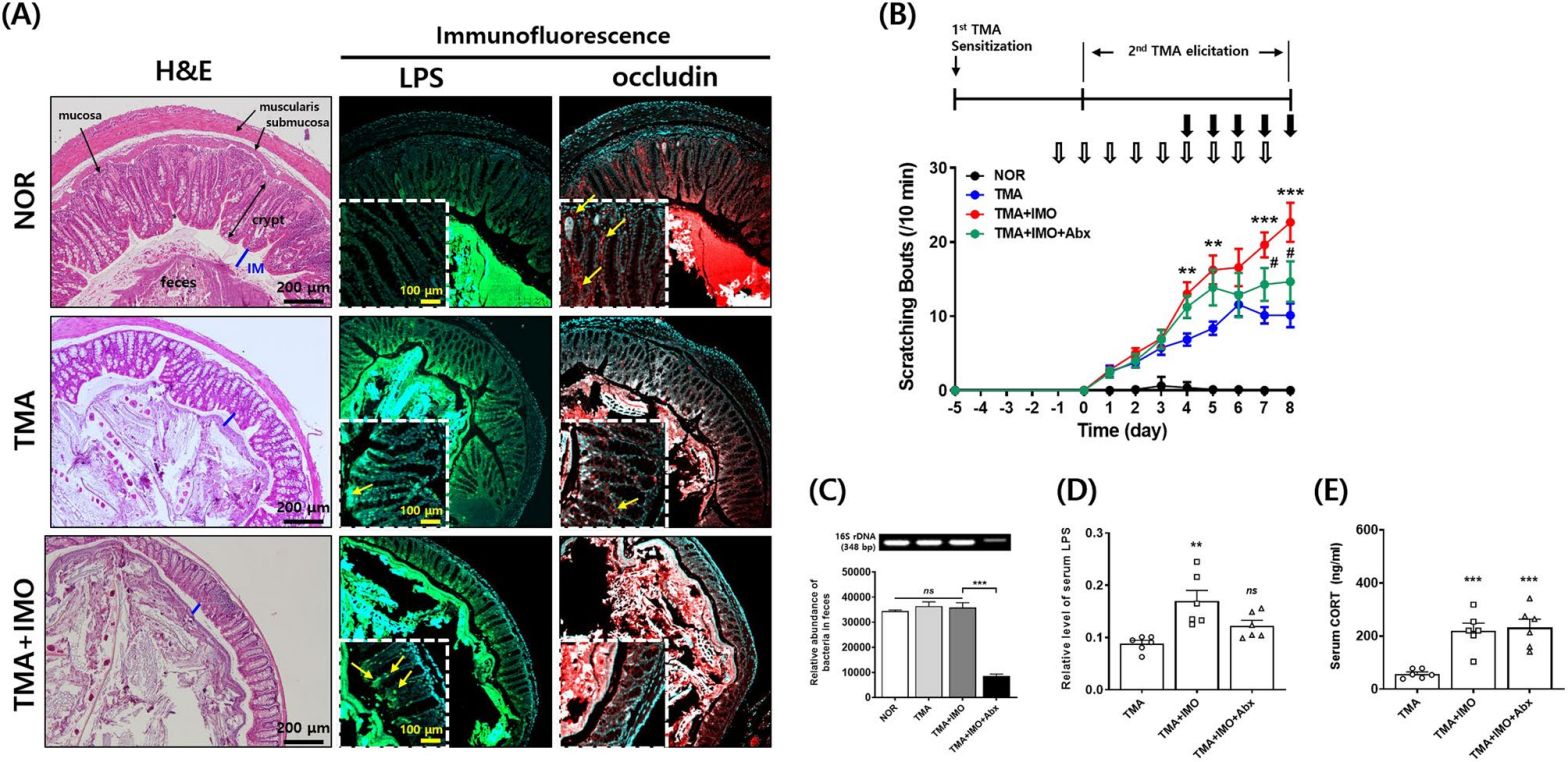
Histological images (**A**) showing the H&E-stained intestinal epithelium and fluorescence-labeled LPS and occludin in paraffin-embedded distal colon sections. In H&E-stained images, blue lines indicate inner mucus layer length. Yellow arrows indicate cells immunopositive for LPS or occludin. Scratching bouts (**B**), the level of total bacteria in the feces (**C**), and serum levels of LPS molecules (**D**) and corticosterone (**E**) were assessed in TMA + IMO-treated mice under antibiotic cocktail (Abx) conditions. Black and white arrows indicate the time points of IMO stress (5 times) and Abx treatment (9 times), respectively. *ns*: not significant. ^**^p < 0·01, ^***^p < 0·001 versus NOR group; *p*^#^ < 0·05 versus TMA + IMO group. IM: inner mucus layer; LPS, lipopolysaccharides; IMO, immobilization; TMA, trimellitic anhydride; NOR, normal; hematoxylin and eosin.

**Figure 4:**
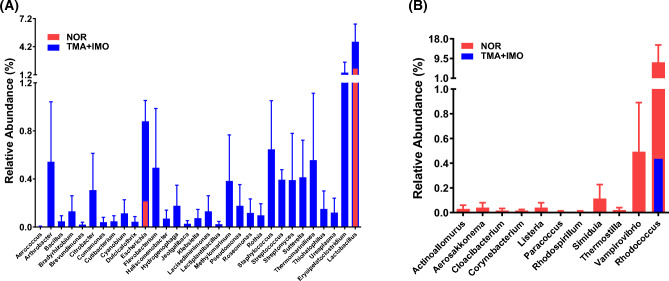
The relative abundance of strict aerobes and facultative anaerobes increased more (or only detected) in the mucus layers of TMA + IMO mice than in NOR mice due to IMO stress (**A**) and in the mucus layers of NOR mice than in TMA + IMO mice (**B**). Red and blue bars indicate bacterial genera that overgrew in NOR and TMA + IMO mice, respectively (n = 3 in each group). Each bar indicates an average value of three mice and is expressed as the mean ± standard error of the mean (SEM). TMA: trimellitic anhydride, IMO: immobilization; NOR, normal.

**Figure 5:**
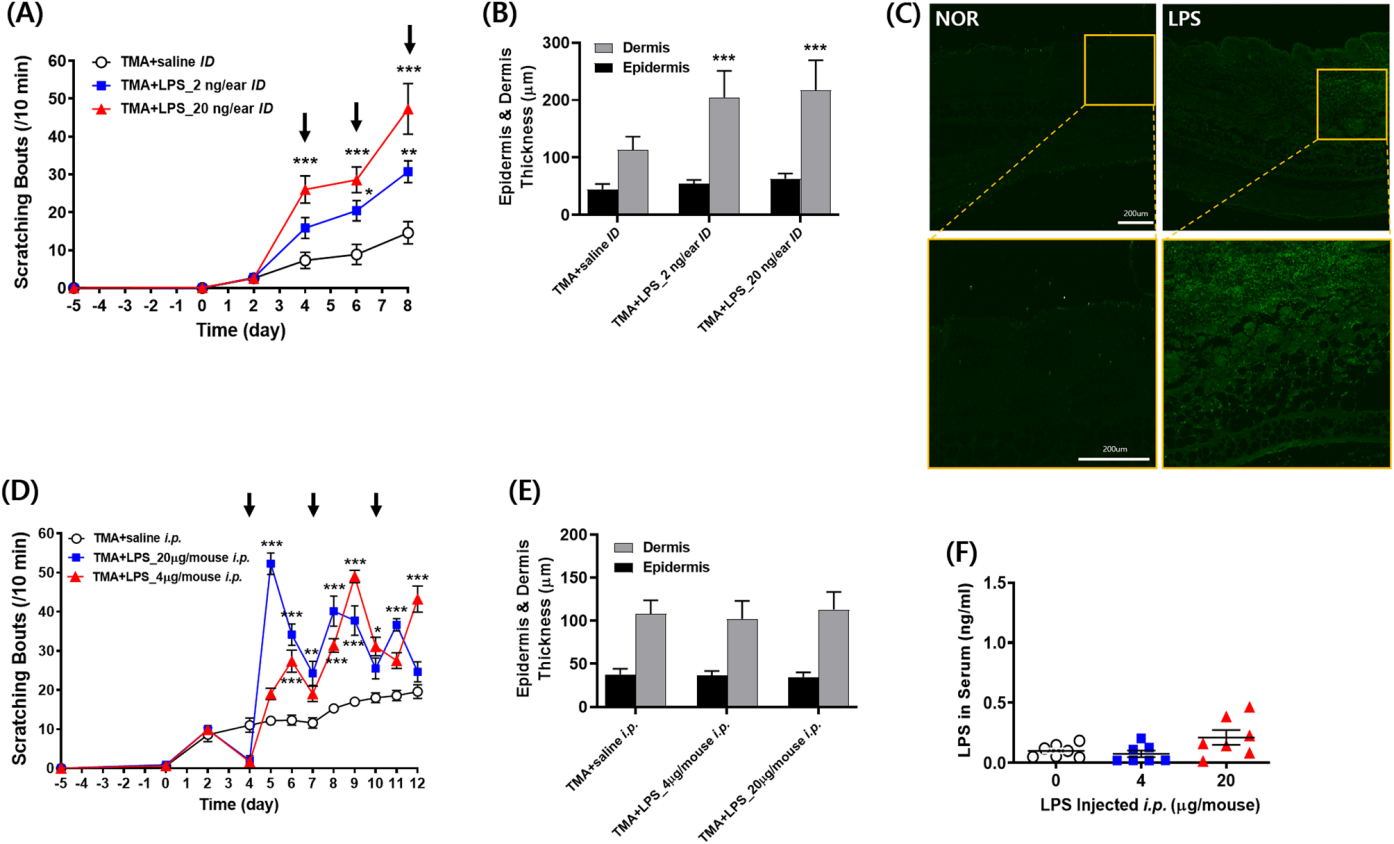
The effects of i.d. and i.p. injections of LPS molecules on the scratching behavior (**A**, **D**) and epidermis and dermis thickness of ear tissue (**B**, **E**), respectively, in TMA-treated mice. I.d.-injected LPS molecules were immunostained in the TMA + LPS_20 ng/ear i.d. group on day 8 (**C**). The serum concentration of i.p. injected LPS molecules was measured in the TMA + LPS_20 μg/mouse i.p. group on day 12 (**F**). Black arrows in A and D indicate the time points of LPS injections. The lower figures in both the NOR and LPS groups depict an enlargement of the orange squares seen in the upper figures. ^*^p < 0·05, ^**^p < 0·01, ^***^p < 0·001 versus TMA + saline i.d. (or i.p.) group. LPS, lipopolysaccharides; TMA, trimellitic anhydride; NOR, normal; i.d., intradermal; i.p., intraperitoneal.

All results are expressed as the mean ± SEM. Statistical differences between groups were identified using one-way analysis of variance (ANOVA) and Tukey’s post hoc test for Fig. 1 B, E, F, G, H, I, J, K, L, M, Fig. 2A,B,D, Fig. 3 B’, B’’,B’’’, and Fig. 5 E’, E’’ (*p* < 0·001, *p* < 0.01 and *p* < 0·05 are represented as *** and ^###^, ^**^ and ^##^, and *, respectively), and two**-**way ANOVA and Bonferroni posttest for Figs. 3 B, 4 A, A’, C, C’, D, and Fig. 5 A, A’, B, B’, C, C’, D, D’, E (*p* < 0·001, *p* < 0·01 and *p* < 0·05 are represented as ***, ^###^ and ^$$$^, ** and ^$$^, and *, respectively). Sample sizes per group are provided in the Supplemental Methods. Differences were considered statistically significant at *p* < 0·05.

### Ethical approval and consent to participate

Animal experiments were performed in accordance with the guidelines for the Care and Use of Laboratory Animals (NIH Publications No. 80-23, revised in 1996) and were approved by the Kyung Hee University Institutional Animal Care and Use Committee (KHSASP-21-089 and KHSASP-22-615).

### Consent for publication

The paper was read and approved by all writers.

## Results

### IMO stress in the elicitation phase of TMA-induced AD exacerbated itch and edema of the mouse ear skin

As neither immobilization stress nor water avoidance stress alone induced skin itch in naïve mice in our preliminary study, the effect of psychological stress on itch sensation was evaluated after the prior onset of allergic contact hypersensitivity in the skin through repeated application of allergic chemicals, such as TMA. The overall experimental procedure is illustrated in Fig. 1A and S1. Scratching behavior began to rapidly increase after the first exposure to IMO stress on day 4, reaching a maximum on day 8, which was five times higher than that of mice treated with TMA only (Fig. 1B: F_2,18_ = 68·82, *p* < 0·001; and C). Histological examination of ear paraffin sections revealed significant increases in edema, indicated by the thickness of the epidermis and dermis of the ear skin in the TMA + IMO**-**treated group (Fig. 1D, E: F_2,18_ = 18·52, *p* < 0·001; F: F_2,18_ = 22·43, *p* < 0·001). In particular, dermal thickness increased more than fivefold by IMO stress in TMA**-**treated mice (Fig. 1F), which was quantitatively consistent with the increasing frequency of scratching behavior. The weights of both submandibular lymph nodes tripled in the TMA**-** and TMA + IMO**-**treated groups compared with those in the normal (NOR) group. However, the difference between the weights of the TMA- and TMA + IMO**-**treated groups was not statistically significant (Fig. 1G: F_2,21_ = 93·10, *p* < 0·001). Moreover, there was no significant difference in the spleen weight between the groups (data not shown).

Subsequently, the responsivity of the hypothalamic‒pituitary‒adrenal (HPA) axis was analyzed by measuring the serum levels of corticosterone (CORT), corticotrophin-releasing hormone (CRH), and adrenocorticotropic hormone (ACTH). Serum CORT levels significantly increased following treatment with TMA compared to those in NOR mice, and it further increased upon exposure to IMO stress by threefold (Fig. 1H: F_2,21_ = 111·5, *p* < 0·001). Serum concentrations of CRH and ACTH, the upstream messengers of the HPA axis, were not significantly different between the TMA- and TMA + IMO**-**treated groups (Fig. 1I, J).

To confirm psychological stress-associated behaviors, an elevated plus maze** (**EPM) test was conducted on day 8 after the final exposure to IMO stress. The anxiety index in the EPM test was notably higher in the TMA + IMO group than in the NOR (vehicle**-**treated) and TMA groups (Fig. 1K). Nevertheless, mice treated with TMA alone did not display a discernible difference compared to vehicle**-**treated mice in the NOR group. This result suggests that repeated treatment with TMA did not induce significant stress in mice, unlike in the TMA + IMO group, in which immobilization stress was additionally applied.

Furthermore, serum levels of proinflammatory type 1 T helper (Th1) cytokines, such as serum interferon (IFN)**-**γ and IL**-**6, were measured to determine whether the intestinal immune response was intensified by treatment with TMA and IMO stress. Both IFN**-**γ and IL**-**6 levels were significantly higher in the TMA**-** and TMA + IMO**-**treated groups than in the NOR group (Fig. 1L: F_2,12_ = 30·66, *p* < 0·001; M: F_2,12_ = 18·27, *p* < 0·001). This increase in Th1 cytokines indicates the manifestation of chronic symptoms of AD**-**like skin dermatitis following the initial phase of the allergic response. However, the difference between the TMA and TMA + IMO groups was not as pronounced as that observed in the parameters of scratching bouts and skin thickness, implying that IMO stress did not significantly affect the humoral Th1 responses to allergic TMA.

### Gut bacterial cells or LPS translocated through the colon epithelial barrier in IMO + TMA-treated mice

To investigate how IMO stress aggravates the itch sensation of the skin and the resulting scratching behavior, intestinal epithelial barrier integrity was examined by analyzing the serum levels of LPS and forcefully ingested fluorescein isothiocyanate (FITC)-dextran. The serum LPS level was significantly increased in the TMA**-**treated group and further increased in the TMA + IMO-treated group (Fig. 2A). However, the increase in serum LPS concentration due to IMO stress did not match the increase in scratching behavior. Colon permeability was also tested using FITC**-**dextran, a fluorescent tracer, to measure intestinal paracellular permeability. Serum levels of FITC**-**dextran administered via oral gavage increased significantly after 4 h on day 8 in the TMA**-**treated group and further increased by IMO stress in the TMA + IMO**-**treated group (Fig. 2B). To demonstrate that the LPS molecules in the blood originated from intestinal bacteria, LPS molecules conjugated with fluorescence dye were traced after being injected into the colon lumen. In the final IMO stress session, Cy 5·5**-**conjugated LPS did not appear on the skin until 4 h after injection, and it was detected in different regions of the dorsal and lateral skin, as well as on the back of the neck, within 10 h (Fig. 2C: F_4,15_ = 11·86, *p* < 0·001; and D).

IMO stress**-**induced deterioration of the colon epithelial structure was also confirmed via histological examination of paraffin-embedded distal colon sections from TMA-treated mice (Fig. 3A). H&E-stained sections showed a degenerated structure of the colon epithelium with blunt and shrunken villi, decreased crypt depth, a thinned mucus layer, and epithelial cells with an uncertain boundary in the TMA + IMO-treated group compared with those in both the NOR and TMA**-**treated groups. Occludin, a tight junction protein responsible for the paracellular passage of molecules, was uniformly expressed on the surface and inside the epithelial cells in the crypts of the NOR group. However, its expression and distribution were significantly downregulated in the TMA**-** and TMA + IMO-treated groups. In NOR mice, LPS molecules were scarcely observed in the villi and crypts, which consisted of a single layer of absorptive columnar cells and goblet cells, as well as in the lamina propria, submucosa, and mucosa of colon sections. However, in the TMA + IMO group, several LPS molecules were observed on the luminal side of the colon epithelium. The quantity of LPS molecules appears to be inversely related to the integrity of the colonic epithelial barrier, crypt depth, and thickness of the inner mucus layer.

If the LPS molecules detected in the blood in the TMA + IMO group originate from intestinal gram-negative bacteria, which translocate across the colon epithelium and aggravate itch sensation in the skin, the elimination of bacterial content in the intestinal lumen should alleviate scratching behavior. To test this hypothesis, TMA + IMO**-**treated mice were orally administered an antibiotic cocktail (Abx) once daily for eight days, from days − 1 to 7, and their scratching behavior was evaluated (Fig. 3B). Abx treatment significantly alleviated scratching bouts; however, the difference between the TMA + IMO-and TMA + IMO + Abx-treated groups was not statistically significant. Scratching bouts in the Abx treatment group did not reach the levels observed in the TMA**-**treated group. Moreover, the levels of total bacteria, assessed by 16S rDNA PCR, in the fecal samples exhibited a notable decrease on day 8 compared to those of the other groups, namely, the NOR, TMA, and TMA + IMO groups (Fig. 3C). In the group of mice treated with TMA + IMO and Abx, there was a marginal decrease in the serum levels of LPS compared to those in mice treated with the vehicle, although this difference was not statistically significant (Fig. 3D). However, there were no discernible differences in the serum levels of corticosterone between the group treated with antibiotics (TMA + IMO + Abx) and the untreated group (TMA + IMO) (Fig. 3E).

### Aerobic and facultative anaerobic bacteria overgrew in the colonic mucus layer, spleen, and mesenteric lymph node in IMO + TMA-treated mice

We hypothesized that LPS in the blood of TMA + IMO-treated mice must be transported from fecal contents across the epithelial gut barrier if bacteria can be isolated from the spleen and mesenteric lymph nodes. Therefore, we first investigated how the bacterial composition and diversity in the mucus layers adhered to colonic epithelial cells were altered by IMO stress in TMA-treated mice using PCR-amplified 16S rRNA gene sequencing data for the identification of bacteria. Interestingly, there were no significant differences in the diversity of gut microbiota in the colon mucus layers between the groups (Fig. S2). Detailed information is provided in the Supplemental Results section.

However, of the 183 identified genera, including two unknown genera, 31 genera showed a higher relative abundance in the TMA + IMO group than in the NOR group or were detected only in the strict aerobes in the TMA + IMO group (15 genera), facultative anaerobes (15 genera), and aerotolerant anaerobes (1 genus) (Fig. 4A). Among them, gram-positive facultative anaerobic gut bacteria, such as *Erysipelatoclostridium* sp. and *Staphylococcus* sp., increased the most in the TMA + IMO group. In contrast, the abundance of only 11 genera, four facultative anaerobes, such as *Cloacibacterium*, *Listeria*, *Rhodospirillum*, and *Thermostilla*, and seven aerobes, such as *Actinoallomurus*, *Aerosakkonema*, *Corynebacterium*, *Paracoccus*, *Simiduia*, *Vampirovibrio*, and *Rhodococcus*, decreased in the TMA + IMO group (Fig. 4B). Taken together, the relative population sizes of many colonic bacteria capable of utilizing oxygen for energy metabolism noticeably increased in the mucus layers under IMO stress conditions.

Bacterial cells translocated through the intestinal epithelial barrier were isolated via anaerobic culture of homogenates of lymphoid organs, such as the spleen and mesenteric lymph nodes, and were identified by colony PCR of 16S rRNA genes and DNA sequencing in the TMA + IMO group (Table 1). Several bacterial colonies were isolated from the spleen and identified as facultative anaerobes, including *Escherichia coli* and *Robertmurraya massiliosenegalensis*, and aerotolerant anaerobes, including *Lactobacillus murinus*. In the case of mesenteric lymph nodes, some facultative anaerobes, such as *Staphylococcus nepalensis*, *R. massiliosenegalensis*, *Bacillus yapensis*, *B. spiralis*, and *B. firmus*, were retrieved using a BLASTN search. The presence of bacteria in organ homogenates was also verified using direct PCR of organ homogenates without cultivation, and more bacteria were identified (Table 1): four facultative anaerobes, including *Streptococcus* sp., *Methylobacterium* sp., *Actinomyces* sp., and *Staphylococcus epidermidis*; five strict aerobes, including *Bosea vestrisii*, *Acinetobacter baumannii*, *Porphyrobacter colymbid*, and *Comamonas denitrificans*; one aerotolerant anaerobe, namely, *Cutibacterium acens*; one strict anaerobe, namely, *Ruminococcus torques* from the spleen; and two strict aerobes, including *Bosea* sp. and *Curvibacter gracilis* from mesenteric lymph nodes. A small number of these bacteria were also detected in the TMA group but not in the NOR group.

**Table 1:** Detection of facultative anaerobic and aerobic bacteria in lymphoid organs under IMO stress conditions: utilizing anaerobic culture and colony PCR, or PCR on organ homogenates in the TMA + IMO group.

Organ	Accession no	BLAST hit	Identity (%)	Oxygen usage	Gram staining	Original habitat (Ref*)
Anaerobic culture of organ homogenates & colony PCR						
Spleen	OM_996123.1	*Escherichia coli*	100	facultative anaerobic	negative	human feces (Méric et al., 2016)
	MT_585453.1	*Lactobacillus murinus*	100	aerotolerant anaerobic	positive	rat feces (Yuan et al., 2020)
	NR_125590.1	*Robertmurraya massiliosenegalensis*	100	facultative anaerobic	positive	human feces (Ramasamy et al., 2013)
Mesenteric lymph node	LC_511695.1	*Staphylococcus nepalensis*	100	facultative anaerobic	positive	oral microbiota of domestic cat (Rossi et al., 2017)
	NR_125590.1	*Robertmurraya massiliosenegalensis*	100	facultative anaerobic	positive	human feces (Ramasamy et al., 2013)
	NR_170510.1	*Bacillus yapensis*	100	facultative anaerobic	positive	deep**-**sea sediment (Xu et al., 2020)
	HM_439461.1	*Bacillus siralis*	100	facultative anaerobic	positive	silage (Pettersson et al., 2000)
	LN_995461.1	*Bacillus firmus*	100	facultative anaerobic	positive	soil and freshwater (Huang et al., 2021)
Direct PCR on organ homogenates						
Spleen	MN_095260.1	*Streptococcus* sp*.*	100	facultative anaerobic	positive	salivary microbiome (Kun et al., 2016)
	MN_606145.1	*Bosea vestrisii*	100	strictly aerobic	negative	hospital water supplies (La Scola et al., 2003)
	AB_910746.1	*Ruminococcus torques*	100	strictly anaerobic	positive	feces of IBS patient (Kassinen et al., 2007)
	CP_054302.1	*Acinetobacter baumannii*	98·21	strictly aerobic	negative	hospital environment (Gootz et al., 2008)
	KM_187436.1	*Sphingopyxis bauzanensis*	100	strictly aerobic	negative	soil (Takeuchi et al., 2001)
	EU_303272.1	*Methylobacterium* sp*.*	99·35	facultative aerobic	negative	soil and water (Omer et al., 2004)
	MN_746242.1	*Porphyrobacter colymbi*	96·40	strictly aerobic	negative	freshwater pond (Fuerst et al., 1993)
	GU_412767.1	*Actinomyces sp.*	100	facultative anaerobic	positive	soil and microbiota of animals (Gajdács et al., 2020)
	MN_372140.1	*Cutibacterium acens*	100	aerotolerant anaerobic	positive	humans' skin (Kristian et al., 2022)
	KU_991392.1	*Comamonas denitrificans*	100	strictly aerobic	negative	activated sludge (Gumaelius et al., 2001)
	ON_013135.1	*Staphylococcus epidermidis*	100	facultative anaerobic	positive	human and mucosal flora (Sabaté Brescó et al., 2017)
Mesenteric lymph node	AB_835775.1	*Bosea* sp.	95·41	strictly aerobic	negative	pyritic shale (La Scola et al., 2003)
	MT_322964.1	*Curvibacter gracilis*	100	strictly aerobic	negative	soil and well water (Ding et al., 2004)

TMA, trimellitic anhydride; IMO, immobilization. *References are described in the Supplementary Materials.

### IMO stress-induced scratching aggravation was reproduced via LPS injection in TMA-treated mice

To confirm whether the exacerbation of scratching due to IMO stress can be attributed to bacterial LPS translocation through the epithelial barrier, TMA-treated mice were intradermally or intraperitoneally injected with a low dose of LPS, which did not cause systemic toxicity. Neither intradermal (i.d.) nor intraperitoneal (i.p.) injection of LPS molecules alone, at doses of up to 20 mg/ear i.d. and 20 μg/mouse i.p., as used in this study, caused scratching behavior in naïve mice. Moreover, mice treated with these doses of LPS did not show signs of sickness owing to LPS toxicity. Direct injection of LPS into the skin of both ears increased scratching bouts in a dose**-**dependent manner (Fig. 5A). Following the first LPS injection on day 4, the behavioral response exhibited a rapid increase, which was further amplified with the second and third injections at both doses compared to that in the saline-injected control in TMA-treated mice. Histological observations of the ear skin revealed a significant increase in dermal thickness but not in epidermal thickness (Fig. 5B). However, no significant differences were observed between the two LPS doses. In contrast to the negligible levels observed in the vehicle-treated NOR group, immunohistochemical analysis revealed a significant presence of LPS in the epidermal layer of ear tissue after i.d. injection (20 ng/ear) (Fig. 5C).

The pattern of increased scratching bouts in response to i.p. injection of LPS differed from that observed after i.d. injection. The initial i.p. injection of LPS led to a sharp increase in scratching bouts the following day, which immediately subsided until subsequent injection. As the number of injections increased, the range of fluctuation in scratching behavior decreased; however, no dose-dependency was observed (Fig. 5D). In contrast to the i.d. injection, no significant difference in dermal thickness was observed between the groups in the i.p*.* injection. (Fig. 5E), and the serum LPS concentration remained unchanged throughout the experiment (Fig. 5F).

### Chemical blockage of colonic permeability alleviated IMO stress-induced aggravation of scratching in TMA-treated mice

To investigate the potential association between the IMO stress-induced increase in scratching behavior and paracellular permeability in the colonic epithelial barrier, three chemical inhibitors of tight junctions were used. These inhibitors include lubiprostone (Lu), which activates chloride channel type 2 (ClC-2) in epithelial cells, promoting water passage into the luminal space and bowel movements^21^. The second inhibitor was 2,4,6-triaminopyrimidine (TAP), which acts as a proton donor that forms double hydrogen bonds with the acid groups of junctional cation channels, thereby abolishing cation permeation. Finally, ML-7, a myosin light chain kinase inhibitor that prevents contraction of the epithelial cell cytoskeleton and subsequent opening of tight junctions, was used^22^.

**Figure 6:**
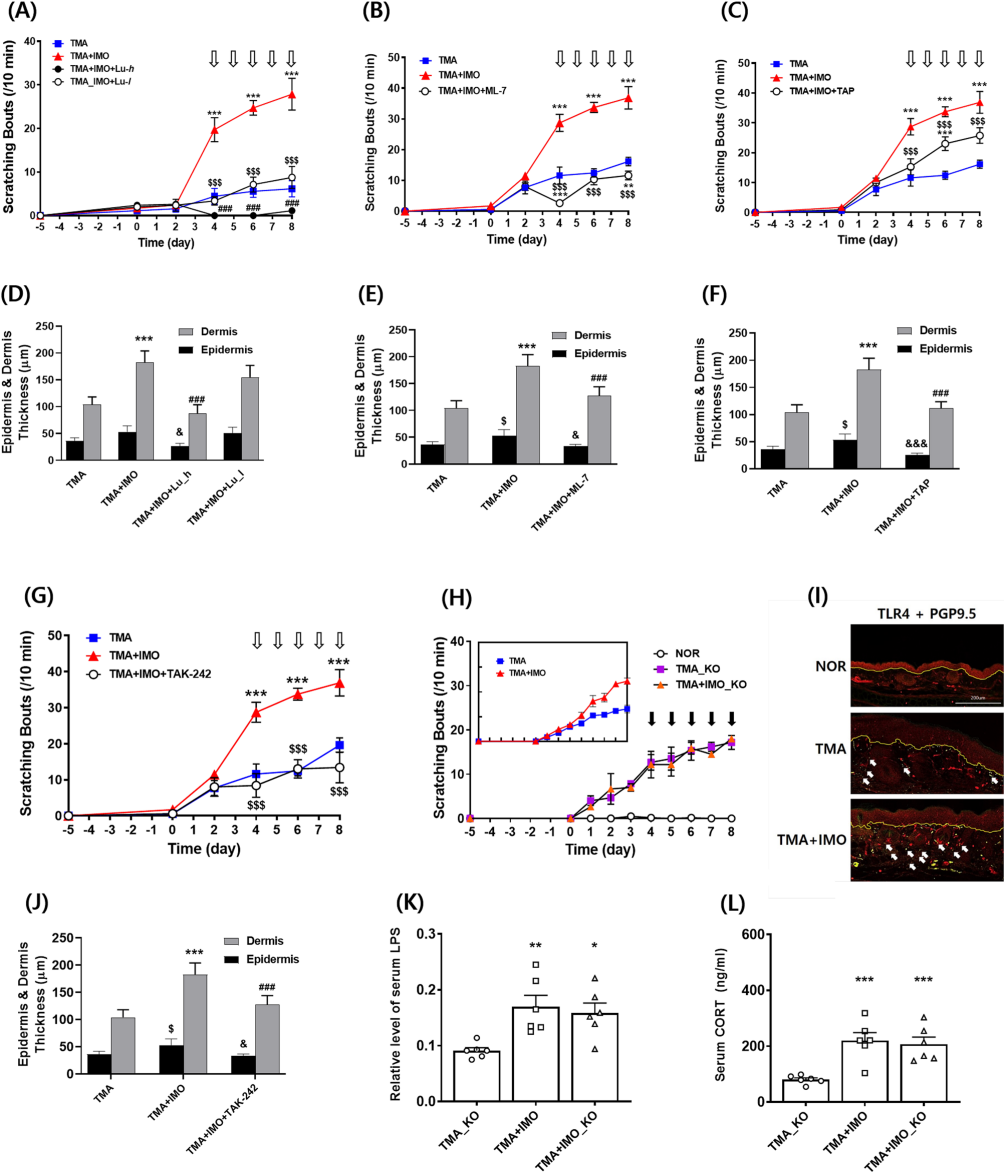
Effects of lubiprostone (Lu), ML-7, TAP, and TAK-242 on scratching behavior (**A**, **B**, **C**, **G**), epidermis, and dermis thickness of ear tissue (**D**, **E**, **F**, **J**), respectively, in the TMA + IMO-treated group. Lu was administered at two different doses: 100 μg/kg in the TMA + IMO + Lu-*h* group and 20 μg/kg in the TMA + IMO + Lu-*l* group. White arrows indicate the time points of inhibitor treatments in A, B, C, and D. Scratching bouts (**H**) and serum levels of LPS (**K**) and corticosterone (**L**) were assessed in TLR4 KO male mice on a C57BL/6 background with TMA or TMA + IMO treatment. The time points of IMO stress (a total of 5 instances) are denoted by the black arrows in E. In the small box in H, scratching behaviors of TMA-and TMA + IMO-treated naïve mice of the C57BL/6 background are presented as a control. TLR4 protein (yellow-stained) in ear tissues was immunostained with PGP9.5 (red-stained, a marker for peripheral nerve fibers) in the TMA + IMO-treated group on day 8 (**I**). White arrows indicate representative immunostained spots of TLR4 proteins in the TMA**-** and TMA + IMO**-**treated groups. The border line between the epidermis and dermis layers is indicated by the yellow lines in F. ^*^p < 0·05, ^**^p < 0·01, ^***^p < 0·001 versus TMA group (or TMA_KO); ^$$$^*p* < 0·001 versus TMA + IMO-treated group. TMA, trimellitic anhydride; NOR, normal; KO, knockout; TLR4, Toll□like receptor 4; IMO, immobilization.

The exacerbation of scratching behavior induced by IMO stress was notably and significantly inhibited in a dose**-**dependent manner by the i.p. injection of Lu (Fig. 6A). At a low dose of Lu (20 μg/mL), the increase in scratching behavior due to IMO stress was completely inhibited, reaching a level equivalent to that observed in TMA-treated mice. Conversely, at a high dose of Lu (100 μg/mL), scratching behavior remained significantly lower than that of TMA**-**treated mice, even under IMO stress. Furthermore, daily injections of ML-7 (1 mg/kg) and TAP (3 mg/kg) during the 5-day treatment period of IMO stress also resulted in significant inhibition of IMO stress-induced increases in scratching behavior (Fig. 6B and C). The inhibitory effect of ML-7 was more pronounced than that of TAP and nearly abrogated the scratching behavior induced by IMO stress in TMA**-**treated mice. The alterations in epidermal and dermal thickness observed in the histological images of ear sections were consistent with the changes in scratching behavior observed in the experiments involving the three types of inhibitors (Fig. 6D, E, and F). Similar to previous results, changes in the dermis were more pronounced than those in the epidermis.

### Selective inhibition of TLR4 signaling and TLR4 knockout extinguished IMO stress-induced aggravation of scratching in TMA-treated mice

To investigate the role of TLR4 in stress-associated aggravation of itch sensation, TAK-242, a small-molecule inhibitor of TLR4 signaling^23^, was intraperitoneally injected 1 h before each IMO stress session for five consecutive days into TMA + IMO**-**treated mice (Fig. 6G). Administration of 2 mg/kg TAK**-**242 completely inhibited the IMO stress**-**induced increase in scratching behavior, and the scratching bouts reached a level similar to that observed in the TMA**-**treated group. Furthermore, the increases in epidermal and dermal thickness were significantly suppressed in the TMA + IMO + TAK-242-treated group compared to those in the TMA + IMO**-**treated group (Fig. 6J).

The role of TLR4 in the stress**-**associated aggravation of itch sensation was further confirmed in TLR4 knockout (KO) mice with a C57BL/6 background. Repetitive treatment with TMA significantly increased scratching behaviors in both TMA_KO (KO mice treated with TMA) and TMA-treated naïve C57BL/6 mice (shown in the box of Fig. 6H) compared to those in the vehicle**-**treated NOR group. However, five exposures to IMO stress had no impact on the scratching behavior of these mice. Consequently, the increasing trend of scratching bouts in the TMA + IMO**-**treated group was similar to that in the TMA**-**treated group of TLR4 KO mice. Collectively, these results indicate that TLR4 signaling plays a pivotal role in stress**-**associated aggravation of itch sensation in TMA**-**treated mice.

Furthermore, in the TMA-treated condition, the introduction of IMO stress to TLR4 KO mice led to a significant elevation in serum levels of LPS (Fig. 6K) and corticosterone (Fig. 6L), similar to levels observed in naïve mice, despite no discernible difference in scratching behavior (Fig. 6H).

In the ear skin tissue, we observed a significant increase in the expression of TLR4 proteins along DRG sensory neurons in the dermal layers in both the TMA and TMA + IMO groups compared with that in the NOR (vehicle**-**treated) group (Fig. 6I). Notably, in the TMA + IMO group, TLR4 expression was further enhanced by IMO stress. These findings strongly suggest that repeated skin exposure to TMA and IMO stress can both contribute to an increase in TLR4 expression in the TMA + IMO group.

## Discussion

The occurrence of various stress**-**related disorders, including autoimmune diseases, psychiatric disorders, and gastrointestinal conditions, including inflammatory bowel disease (IBD) and IBS, is closely linked to the composition of the intestinal microbiota, integrity of host barrier function, and mucosal immune response^24–28^. These factors are significantly influenced by the complex interaction between intestinal epithelial cells and luminal contents, including the gut microbiota. Therefore, maintaining the integrity of intestinal permeability is crucial for achieving a balance between protective and tolerogenic immune responses involving the gut microbiota and antigenic food molecules^6,29,30^. When the delicate equilibrium is disrupted, various illnesses associated with impaired gut detection of harmful microorganisms may arise or worsen^31,32^. These conditions include stress-induced IBS, a functional gastrointestinal disorder, and stress-induced exacerbation of IBD, an inflammatory condition affecting the gastrointestinal tract^6,33^. Although IBS and IBD are distinct gastrointestinal disorders with varying pathologies and causes, they share specific overlapping symptoms such as abdominal pain and discomfort. Notably, both conditions are strongly associated with emotional stress and often exhibit extraintestinal manifestations on the skin, such as psoriasis, pruritus, and AD, which are also strongly linked to stress.

In the present study, we observed that bacterial cells or their antigenic LPS molecules originating from the gut lumen could breach the compromised colonic epithelial barrier under IMO stress. Subsequently, these penetrated particles are transported through the blood or lymphatic vessels to the skin, influencing itch sensation via interactions with sensory neurons^14^*.* Our findings indicated that LPS molecules exacerbated itch sensation through TLR4, a receptor that is more abundant or activated in the skin or on a specific subset of DRG sensory neurons in patients with AD and/or AD animal models^34,35^. In our study, this upregulation seemed to occur because of repeated exposure to antigenic molecules such as TMA. Notably, psychological stress alone, such as water avoidance or restraint stress, did not induce scratching behavior or skin edema in either naïve NC/Nga or BALB/c mice when they were maintained under conventional housing conditions (unpublished).

The stress**-**induced increase in scratching bouts was observed only after the onset of TMA**-**induced itch^36^. This observation suggests that the exacerbation of itch sensation occurred independently of the initial itch generation mechanism, irrespective of whether it was histaminergic or nonhistaminergic. Similarly, neonatal maternal separation stress also exacerbates colonic permeability and colitis in naturally occurring IBD in IL-10 KO mice, but this psychological stress alone did not induce colitis in naïve mice^37^. In another report, intracerebroventricular corticotrophin releasing factor (CRF) administration inhibited scratching induced by histamine or chloroquine via CRF receptor 2, shedding light on how acute stress can have anti-itch properties^38^. In addition, in the present study, itch aggravation by psychological stress might have needed a sufficient duration of TMA exposure, involving two immunologic phases of sensitization and elicitation, to activate resident immune cells such as Th1, Th2, and/or Th17 cells. Given the highly pruritic nature of this contact hypersensitivity sensitization reaction, it appears that somatosensory neurons are also modulated during this period and contribute to itch transmission.

TLR4-KO mice experience a significant decrease in skin itching induced by topical application of histamine or chloroquine^14^, suggesting that TLR4 signaling plays a role in the exacerbation of itching associated with mental stress in the skin. In our study, we investigated the role of intestinal LPS molecules in the worsening of scratching behavior. To establish its involvement, we induced itch aggravation through LPS injection instead of IMO stress. Furthermore, we could mitigate this aggravation by administering inhibitors of intestinal paracellular permeability and TLR4 signaling. Additionally, we observed that TLR4-KO mice did not exhibit itch aggravation, indicating an essential role of TLR4 in this process. In addition, intradermal injection of LPS immediately increased scratching bouts on the injection day. However, when LPS was intraperitoneally injected, an increase in itch was observed the following day and persisted for a short period, after which a sharp decrease in scratching bouts was observed. Notably, on the day of i.p. LPS injection, scratching bouts decreased, which could be explained by LPS toxicity, despite the administration of a relatively low dose. This decrease in scratching behavior may also be influenced by the dilution of LPS through the blood circulation.

Notably, the injection of LPS as a replacement for IMO stress did not result in the same level of ear edema, as evidenced by ear thickness in TMA-treated mice, indicating that IMO stress has additional effects beyond merely enhancing itch signaling. These effects may involve interactions with other luminal molecules, such as viral and fungal components, their metabolites, and nondigestible food ingredients, the resulting recruitment of immune cells into skin tissues, and the secretion of inflammatory cytokines, all of which contribute to the observed ear edema. Presumably, the more comprehensive intestinal immune system also appears to be activated through various TLRs located in the lamina propria of the mucosa and submucosa in response to a range of luminal components, such as viruses, fungi, undigested food, and LPS molecules^39,40^. This assumption was supported by the increased serum levels of IFN-γ and IL-6 after five instances of IMO stress. Therefore, it is plausible to propose that during periods of stress, bacteria, their LPS molecules, and other luminal contents can breach the intestinal barrier and enter the bloodstream and lymph vessels. Subsequently, they might disseminate to peripheral skin tissues and bind to their specific TLRs on sensory neurons responsible for itch perception^14^. This interaction could amplify the itch signals formally triggered by the allergenic chemical TMA, as observed in our study. In addition, this process may be facilitated by the excitation of TRPV1 or TRA1 receptors^41^.

Considering the significant role of bacterial LPS in the exacerbation of atopic itch during stress in our study, it is important to note that stress-induced changes in the composition of the intestinal microbiota and the expansion or reduction of specific bacterial strains or populations are not the sole contributing factors. In the TMA + IMO group, there was a notable expansion of oxygen-utilizing bacteria, including strict aerobes and facultative anaerobes, within the colon mucus layers compared to that in the NOR group. A substantial majority of bacteria were isolated from the spleen and mesenteric lymph nodes in the TMA + IMO group and identified as strict aerobes or facultative anaerobes, except for *Ruminococcus torques*, a typical anaerobic commensal bacterium in human fecal matter, as indicated in Table 1. Notably, a shift from obligate anaerobic to facultative anaerobic bacteria has been observed in patients with chronic conditions such as colorectal cancer, obesity, diabetes, IBD, cardiovascular diseases, and neurodegenerative diseases^42,43^. This shift is attributed to the disruption of epithelial hypoxia, leading to increased oxygen levels emanating from the colonic epithelia. As a result, the intestinal environment becomes highly oxygenated, creating favorable conditions for the proliferation of aerobic and facultative aerobic bacteria. In a healthy intestine, the proliferation of oxygen-utilizing bacteria, including strict aerobes and facultative anaerobes, is constrained, whereas the dominant gut microbial population primarily consists of strictly anaerobic bacteria. These anaerobic bacteria ferment dietary fibers within the intestine, leading to the production of butyrate, which in turn activates peroxisome proliferator-activated receptor (PPAR)-γ signaling in colonocytes, facilitating the aerobic metabolism of mitochondrial β**-**oxidation. This process is crucial for maintaining epithelial hypoxia^44^. Hence, the proliferation of aerobic and facultative anaerobic bacteria can serve as a characteristic feature of colon dysbiosis, initiating a feedback loop that disrupts the aerobic metabolism of colonocytes. In summary, these findings suggest that gut bacteria, which have a preference for or the ability to thrive and utilize oxygen for their growth, migrate toward the colonic epithelial barrier and subsequently establish residence within the mucus layer in the TMA + IMO group. Furthermore, it is likely that these bacteria translocate across the epithelial barrier via the paracellular route, possibly due to psychological stress.

**Figure 7:**
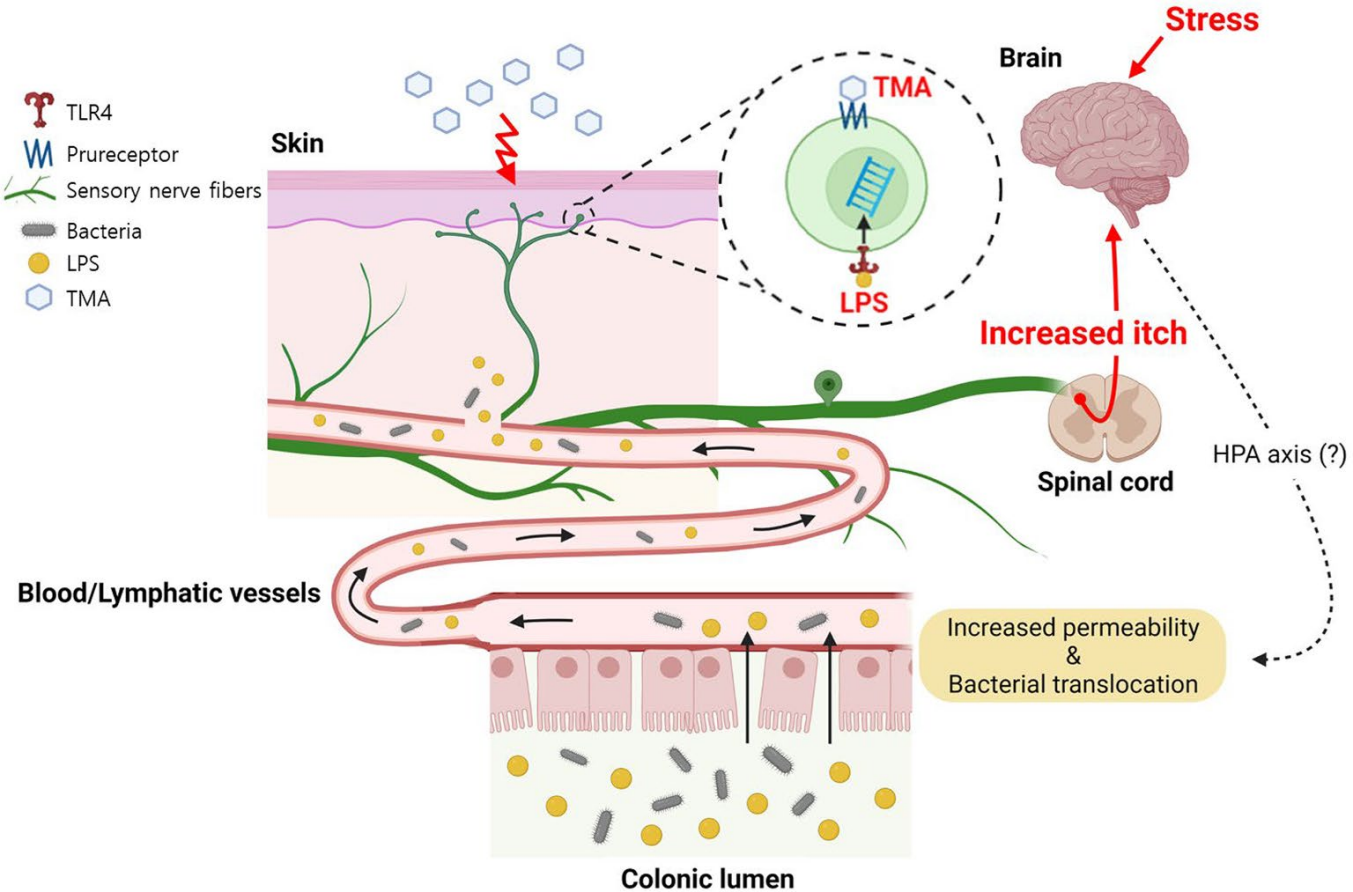
A proposed mechanism of translocation of gut-derived bacterial LPS molecules across intestinal epithelium and the peripheral activation of TLR4 in IMO stress-induced itch aggravation in a mouse model of TMA-induced AD (created with BioRender.com).

While previous studies have predominantly focused on changes in the intestinal immune system and skin barrier function, the connection between these two distant organs has remained unexplored^45,46^. To the best of our knowledge, this is the first in-depth investigation to experimentally demonstrate the involvement of gut bacteria or their components, such as LPS, in the exacerbation of itch sensation under stress conditions in an AD murine model. Figure 7 illustrates a schematic representation of the pathomechanism involving the translocation of gut-derived bacteria and bacterial LPS molecules through the intestinal epithelium, their subsequent journey via the bloodstream, and the resulting activation of TLR4, likely on sensory neurons. This mechanism is associated with the exacerbation of itching in an AD mouse model under IMO stress. Nevertheless, this study had certain limitations and areas that require further exploration. For instance, there is a need to investigate the functions of bacterial components beyond LPS, pinpoint the specific peripheral locations of bacterial LPS molecules in relation to neuronal TLR4 signaling, and uncover the underlying mechanisms governing stress-associated molecules, such as CRF, ACTH, cortisol, and norepinephrine, which are responsible for dysfunction of the gut epithelial barrier.

Additional research is essential to shed light on the mechanisms by which the neurochemical aspects of the stress circuit influence the composition of the gut microbiota, contributing to the disruption of paracellular permeability within the intestinal epithelial barrier. Further investigations are warranted to understand how emotional stress impacts the energy metabolism of colonocytes, both independently and in conjunction with the gut microbiota, despite the substantial difference in gut microbiota composition. Furthermore, careful consideration is needed regarding the potential extrapolation of our experimental findings from mice to humans. Although there has been a report indicating a high frequency of the TLR-2 R753Q single nucleotide polymorphism (SNP) among adult atopic dermatitis patients^14^, it remains uncertain whether this mutation or TLR-2 stimulation with gram (+) bacterial ligands influences the pathogenesis of atopic dermatitis. In rodent models of skin pruritus, pattern-recognition receptors such as TLR 3, 4 and 7, which detect viral genomic RNA and bacterial LPS, have been observed to be expressed in itch-specific DRG sensory neurons. It will be crucial to verify in future studies whether these receptors are similarly expressed in human AD patients, and whether their activation by endogenous and exogenous pathogen-derived molecules could potentiate itch signaling in the skin.

## Conclusions

Our findings indicate that that stress contributes to gut dysbiosis, resulting in the proliferation of oxygen-utilizing bacteria, including obligate anaerobes and facultative anaerobes, within the mucosal layers of the colon epithelium. This stress-driven dysbiosis promotes the migration of these bacteria and their components, including LPS, through the damaged colon epithelial barrier. Consequently, this process significantly activates peripheral TLR4 signaling and exacerbates skin itchiness. Thus, it is evident that gut dysbiosis and increased intestinal permeability play a pivotal role in the stress-associated aggravation of skin itchness, often observed in adult AD patients. Importantly, this aggravated itch sensation occurs independently of the initial itch generation mechanism in hapten-induced mouse models of AD.

### Supplementary Information

Supplementary Information.

## Data Availability

All data and materials are available in the main text or the supplemental materials.

## Abbreviations

AD: Atopic dermatitis
IBS: Irritable bowel syndrome
LPS: Lipopolysaccharides
Cy5.5: Cyanine 5.5
TLR: Toll**-**like receptor
TMA: Trimellitic anhydride
HPA: Hypothalamic‒pituitary‒adrenal
CORT: Corticosterone
CRH: Corticotrophin-releasing hormone
ACTH: Adrenocorticotropic hormone
i.d.: Intradermal
i.p.: Intraperitoneal
i.c.: Intracolonic
KO: Knockout
H&E: Hematoxylin and eosin
ML**-**7: 1**-**(5**-**Iodonaphthalene**-**1**-**sulfonyl)**-**1H**-**hexahydro**-**1,4**-**diazepine hydrochloride
MLCK: Myosin light chain kinase
TAP: 2,4,6**-**Triaminopyrimidine
FITC: Fluorescein isothiocyanate
