# Supplementary material for: Role of gut-derived bacterial lipopolysaccharide and peripheral TLR4 in immobilization stress-induced itch aggravation in a mouse model of atopic dermatitis
Source: Sci Rep. 2024 Mar 15;14:6263. doi: 10.1038/s41598-024-56936-z (PMC10942979; doi:10.1038/s41598-024-56936-z)
Supplement: Supplementary file 1 — Supplementary Information. [file 41598_2024_56936_MOESM1_ESM.docx]

**Supplementary Materials for**

**Role of gut−derived bacterial lipopolysaccharide and peripheral TLR4 in immobilization stress-induced itch aggravation in a mouse model of atopic dermatitis**

Hahm et al*.*

*Corresponding author. Email: [dhhahm@khu.ac.kr](mailto:xxxxx@xxxx.xxx)

**This file includes:**

Supplementary Methods

Supplementary Results

Figures S1 and S2

Additional Materials (Original PCR image)

**Supplemental Methods**

**Study design**

Nine independent animal cohorts were used in this study (Fig. S1). Most of the animal studies were conducted with BALB/c mice, chosen for their suitability in inducing TMA (a hapten)−induced contact hypersensitivity on the skin, serving as a chronic model for atopic dermatitis (AD) (Schneider et al., 2009). However, for the experiment on the aggravation of scratching behavior in TLR4 knockout (KO) mice, we used C57BL/6 mice that were already produced and supplied. Animals were randomly assigned to different cohorts using a random number generator. In **cohort 1**, 10−week−old BALB/c male mice were randomly assigned into four groups (n = 8 per group) as follows: vehicle−treated normal group (NOR), TMA−treated group (TMA), and TMA− and IMO−treated group (TMA+IMO). They were subjected to six scratching behavior tests during the TMA and IMO stress treatments over 13 days. On the last day of IMO stress (day 8), serum, feces, lymph nodes, spleen, and colon and ear tissues were harvested after the elevated plus maze (EPM) test for further experiments. In **cohort 2**, which is the same as cohort 1, the FITC−dextran assay was performed on day 8. In **cohort 3**, intradermal (i.d.) injection of LPS into ear tissues was assessed in TMA−treated mice. Ten−week−old BALB/c male mice were randomly assigned into three groups (n = 7 per group) as follows: vehicle (TMA+saline ID), 2 ng LPS−*ID* injected (TMA+LPS_2 ng/ear *ID*), and 20 ng LPS−*ID* injected (TMA+LPS_20 ng/ear *ID*) groups. They were subjected to six scratching behavior tests, and ear tissues were harvested on day 8. In **cohort 4**, intraperitoneal (i.p.) injection of LPS was assessed in TMA−treated mice. Ten−week−old BALB/c male mice were randomly assigned into three groups (n = 7 per group) as follows: vehicle (TMA+saline i.p*.*), 4 mg LPS−i.p. injected (TMA+LPS_4 mg/mouse *i.p.*) and 20 mg LPS**-**i.p*.* injected (TMA+LPS_20 mg/mouse i.p*.*) groups. They were subjected to 12 scratching behavior tests, and ear tissues and serum were harvested on day 12. In **cohort 5**, i.p*.* injection of lubiprostone (low and high doses) was performed in TMA**-**treated mice. Ten−week−old BALB/c male mice were randomly assigned into four groups (n = 7 per group) as follows: control group (TMA), TMA− and IMO−treated group (TMA+IMO), 20 mg/kg lubiprostone**-**treated group (TMA+IMO+Lu−*l*), and 100 mg/kg lubiprostone−treated group (TMA+IMO+Lu−*h*). They were subjected to six scratching behavior tests, and ear tissues were harvested on day 8. In **cohort 6**, treatments with ML−7 hydrochloride, 2,4,6 triaminopyrimidine (TAP), and TAK−242 (resatorvid) were conducted in TMA−treated mice. Ten−week−old BALB/c male mice were randomly assigned into four groups (n = 7 per group) as follows: control group (TMA), TMA− and IMO−treated group (TMA+IMO), 1 mg/kg ML−7−i.p*.* injected group (TMA+IMO+ML**-**7), 3 mg/kg TAP−intracolonic (i.c*.*) injection group (TMA+IMO+TAP), and 2 mg/kg TAK−242−i.p*.* injected group (TMA+IMO+TAK−242). They were subjected to six scratching behavior tests, and ear tissues were harvested on day 8. In **cohort 7**, the effect of IMO stress on scratching behavior was assessed in TMA−treated TLR4 KO mice. Nine−week−old C57BL/6 male mice were randomly assigned into three groups (n = 6 per group) as follows: vehicle−treated normal group (NOR), TMA−treated KO group (TMA_KO), and TMA− and IMO−treated KO group (TMA+IMO_KO); they were subjected to ten scratching behavior tests, and serum was harvested on day 8. In **cohort 8**, translocation of Cy5·5−labeled bacterial LPS (NANOCS, New York, NY, USA) was conducted in nude mice after TMA and IMO stress treatments. Ten−week−old BALB/c nude male mice were randomly assigned into two groups (n = 4 in each group) as follows: vehicle-treated normal group (NOR) and TMA− and IMO−treated group (TMA+IMO). On the last day (day 8) of the daily IMO stress period for 5 days, nude mice were anesthetized with inhaled isoflurane. After a midline laparotomy, the rectum was isolated between silk ties. Cy5·5−labeled bacterial LPS (0·15 mg per mouse) was directly injected into the proximal colon of the isolated intestine. The abdominal wall was then closed with silk sutures. After the operation, *in vivo* images were time−dependently captured. In **cohort 9**, ten−week−old BALB/c male mice were randomly assigned into four groups (n = 8 per group) as follows: vehicle−treated normal group (NOR), TMA−treated group (TMA), TMA− and IMO−treated group (TMA+IMO), and TMA+IMO− and antibiotic−treated group (TMA+IMO+Abx). They were subjected to ten scratching behavior tests during the treatments of TMA and IMO stresses over 13 days. On the last day of IMO stress (day 8), serum was harvested.

**Cultural analysis**

A vinyl anaerobic chamber (SeouLin Bioscience, Seongnam−si, Republic of Korea) containing a gas mix of 5% CO_2_, 5% H_2_, and 90% N_2_ (Daehan Special Gas Co., Siheung−si, Gyeonggi**-**do, Republic of Korea) was used to manipulate organs for cultivation. The whole spleen or lymph node was mashed with iris scissors (Professional Hospital Furnishers, Sialkot, Pakistan) in 1 mL of reinforced clostridial agar (RCA) broth (BD Biosciences, Franklin Lakes, NJ, USA) in an anaerobic incubator at 37 ℃ for 3 to 4 days. Each organ homogenate of 200 μL was spread on an RCA agar plate and incubated in an anaerobic incubator at 37 ℃ for up to 7 days. Next, single colonies on the agar plate were picked using an inoculating loop (SPL Life Sciences, Pocheon−si, Gyeonggi−do, Republic of Korea) and streaked on a new RCA agar plate. Single colonies on the second plate were then used for colony PCR and 16S rRNA sequencing for bacterial identification. PCR primer sequences and operating conditions were as follows: total bacteria (66 ℃, 348 bp) Forward (F) 5′**-**TGGCTCAGGACGAACGCTGGCGGC**-**3′, Reverse (R) 5′−CCTACTGCTGCCTCCCGTAGGAGT−3′; (Yeom et al., 2015) full−length 16 s rRNA gene (58 ℃, 1500 bp) F 5′−AGAGTTTGATYMTGGCTCAG−3′, R 5′**-**ACGGYTACCTTGTTACGACTT−3′, V3−V5 (50 ℃, 587 bp) F 5′−CCTACGGGAGGCAGCAG−3′, R 5′−CCGTCAATTCMTTTRAGT−3′ (Darwish et al., 2021). Direct PCR and 16S rRNA sequencing were also performed using chromosomal DNA samples purified from spleen or lymph node homogenates. The frozen organs were homogenized with a long−type SK−Mill 200 (Diagnocine, Hackensack, NJ, USA). Chromosomal DNA was extracted using a SPINeasy DNA kit (MP Biomedicals, Irvine, CA, USA) according to the manufacturer’s instructions. Following PCR of V3**-**V5, proper target PCR bands were isolated from 1·0% agarose gels stained with GelRed^®^ (Biotium, Fremont, CA, USA) and sequenced. After direct sequencing of the PCR product on the gel, taxonomic assignments were performed using BLASTn against the NCBI 16S microbial database.

**16S rRNA sequence−based microbiota analysis**

Changes in the gut bacterial composition in the colonic mucus layers of naïve and IMO**-**stressed mice were analyzed by sequencing the bacterial 16S rRNA gene. The mucus layer was collected according to the protocol of Johan Dicksved et al. (2021). Briefly, a colonic segment 1.0−1.5 cm away from the rectum was cut from the mouse intestine and opened longitudinally. The luminal contents were gently removed using forceps without scraping the surface. The remaining contents on the colonic tissue sheet were picked away until no visible particles remained. A pipette tip was linked to the soft tube of a vacuum pump. Using a gentle vacuum, the mucus was sucked into the tip. By pipetting, mucus was transferred to Eppendorf tubes containing 100 μL of PBS, quickly frozen in liquid nitrogen, and stored at −80 °C until DNA extraction. Microbial DNA was extracted using a DNeasy PowerSoil Pro Kit (Qiagen, Hilden, Germany) following the manufacturer’s instructions. The V3**–**V4 hypervariable region of the 16S rRNA gene was amplified by PCR using the 341F (5′−CCTACGGGNGGCWGCAG−3′) and 805R (5′−GACTACHVGGGTATCTAATCC−3′) barcoded primers and sequenced using the Illumina MiSeq platform (Illumina, San Diego, CA, USA). Reads were screened for low-quality bases and short read lengths, assembled, and assigned to operational taxonomic units (OTUs) with a similarity threshold of 97%. Taxonomic assignments were performed using BLASTn against the NCBI 16S microbial database. The α− and β−diversity analyses were performed using the QIIME tool (v 1.9). Alpha diversity was analyzed by the Chao 1, observed OTU, and Shannon indices. Beta diversity was determined using weighted UniFrac phylogenetic distance matrixes visualized in principal coordinates analysis (PCoA) plots and tested for statistical significance by analysis of similarities (ANOSIM) with 999 permutations. DESeq2 in QIIME was used to determine significant differences (*p* < 0·05) in OTU bacterial relative abundance.

**Elevated plus maze (EPM) test**

To assess the response to a novel stressful environment, such as TMA treatment and/or IMO stress, mice were subjected to the EPM tests as described, with minor modifications. Briefly, a test mouse was placed in the center of the maze facing an open−arm, and behavior was video-recorded in dim red light (2 × 60 W). The numbers of open-arm entries and closed−arm entries and the time spent in open arms were recorded during the 5−min test period using a video camera mounted on the ceiling above the center of the maze with the SMART program (PanLab, Barcelona, Spain). Open-arm entries were presented as % of the total number of entries. Entry into an arm was defined as placing four paws within a particular arm. The number of fecal pellets was recorded to estimate stress**-**induced defecation. The anxiety index was calculated as follows:

$$\text{the }\text{Anxiety index}\text{ = 1 -}\left[ \frac{\left( \frac{\text{open arms cumulative duration}}{\text{total test duration}} \right)\text{ + }\left( \frac{\text{open arms entries}}{\text{total number of entries to closed arms + open arms}} \right)}{\text{2}} \right]$$

**Immunofluorescence staining**

Sections were deparaffinized and rehydrated using graded ethanol solutions before undergoing antigen retrieval. Slides were immersed in antigen retrieval buffer using sodium citrate tribasic dihydrate (S4641; Sigma−Aldrich) and heated in a microwave oven for 5 min. The slides were then rinsed in 1× PBS at room temperature for 20 min. Sections received 0·3% H_2_O_2_ solution dropwise, followed by incubation at RT for 15 min. Subsequently, sections were washed with 1X PBS three times for 5 min each and blocked in 10% goat normal serum (Vector Laboratories, San Francisco, CA, USA) in 1X PBS for 2 h. Primary monoclonal antibodies of occludin (OC−3F10 [1:100], Thermo Fisher Scientific Cat# 33-1500, RRID: AB_253310; Waltham, MA, USA), LPS [1:50] (Cat# PA1-73178, RRID:AB_1017872), and TLR4 [1:50] (Santa Cruz Biotechnology Cat# sc−293072, RRID:AB_10611320, Dallas, TX, USA) were added, and the sections were incubated at 4 ℃ overnight. After washing, the sections were incubated with secondary antibodies conjugated to fluorescence dyes, such as Alexa Fluor^TM^ 568 (Thermo Fisher Scientific Cat# A−11004, RRID:AB_2534072, Goat), 488 (Thermo Fisher Scientific Cat# A−11059, RRID: AB_ 2534106, Rabbit), and 647 (Thermo Fisher Scientific Cat# A−31571, RRID:AB_162542, Donkey), at RT for 2 h in the dark. Finally, the sections were mounted on coverslips with VAETASHIELD^®^ with DAPI Mounting Medium (Vector Laboratories) and examined using a confocal laser scanning microscope (Fluoview FV10i; Olympus Co.). Objective lens magnification 10X; depth of field: 300 μm.

**Supplementary Results**

**Influence of IMO stress on the gut microbiota adhered to the colon mucus layer in TMA−treated mice**

To investigate the potential impact of IMO stress, which exacerbated itch sensation in TMA−treated mice with AD**-**like skin symptoms, on the intestinal microbiota, the gut microbiota of each experimental group was analyzed using 16S rRNA gene sequencing. Considering the importance of mucus−associated microbiota and its relevance to mucus**-**gut microbiota interactions and bacterial translocation across the epithelial barrier, samples containing the microbiota were harvested from the mucus layer adhering to the epithelial cells of the colon rather than from fecal matter within the colon lumen (Li et al., 2015). There were no significant differences in α−diversity, which was analyzed by the observed OTU, Chao 1, and Shannon indices, between groups (Fig. S2A–C). In addition, PCoA did not exhibit significant differences for either weighted or unweighted UniFrac distance metrics (Fig. S2; ANOSIM: *p* = 0·46, R = 0·021 and *p* = 0·31, R = 0·078 for unweighted (D) and weighted (E) analysis, respectively) between groups. However, at the phylum level, the relative abundance of Bacteroidetes was lower in the TMA+IMO group than in the NOR and TMA groups despite no significant difference in either the a**-** or β-diversities. Conversely, there was a noticeable increase in the abundance of the Firmicutes phylum in the TMA+IMO group (Fig. S2F). Furthermore, the TMA+IMO group demonstrated a discernibly distinct pattern compared to the remaining two groups, whereas the patterns observed in the NOR and TMA groups were remarkably similar. These findings suggest that psychological stress has a greater impact on the composition of the gut microbiota than exposure to TMA, which is known to induce AD−like skin symptoms and scratching behavior. Notably, in the TMA+IMO group, there was a significant increase in the proportion of microbial taxa categorized as ‘others’, which had a relative abundance lower than 0.02%, compared to the NOR and TMA groups.

At the genus level, a significant difference in the relative abundance of mucus−associated microbiota was observed between the TMA+IMO group and the other two groups (NOR and TMA) (Fig. S2G). In the TMA+IMO group, there was a substantial increase in the proportion of microbial taxa categorized as ‘others’, which had a relative abundance lower than 0.02%. Additionally, the relative abundance of *Muribaculum* sp., an important commensal obligatory anaerobic bacterium, was markedly reduced in the TMA+IMO group compared with that in the NOR and TMA groups. No such difference was observed between the NOR and TMA groups, indicating that the IMO**-**induced stress specifically affected the relative abundance of *Muribaculum* sp., whereas TMA treatment alone did not.

The abundance of *Muribaculum* sp. in the mouse gut microbiota suggests that psychological stress induced by IMO can disrupt anaerobic homeostasis in the intestine (Lagkouvardos et al., 2019). This finding aligns with another study involving wild−type mice treated with dextran sulfate sodium (DSS) and housed with Dusp6 KO mice, which enhances colonic barrier integrity and maintains an anaerobic environment conducive to obligatory bacteria. In that study, an increase in the abundance of *Muribaculum* bacteria at the genus level was observed (Chang et al., 2021).

The decrease in the abundance of *Muribaculum* sp. observed in the TMA+IMO group due to psychological stress could be associated with weakening tight junctions, leading to reduced colonic barrier integrity. Conversely, this reduction in *Muribaculum* sp. could interfere with the anaerobic conditions necessary for the survival of other obligatory anaerobic bacteria in the intestinal ecosystem by disrupting the regular mitochondrial β−oxidation process in colon epithelial cells, as reported in previous research (Litvak et al., 2018). The observed reduction in the relative abundance of the Bacteroidetes phylum in Fig. S2G can likely be attributed to the decrease in *Muribaculum* sp., given its taxonomic affiliation within the Bacteroidetes phylum.

**Supplemental Figures**


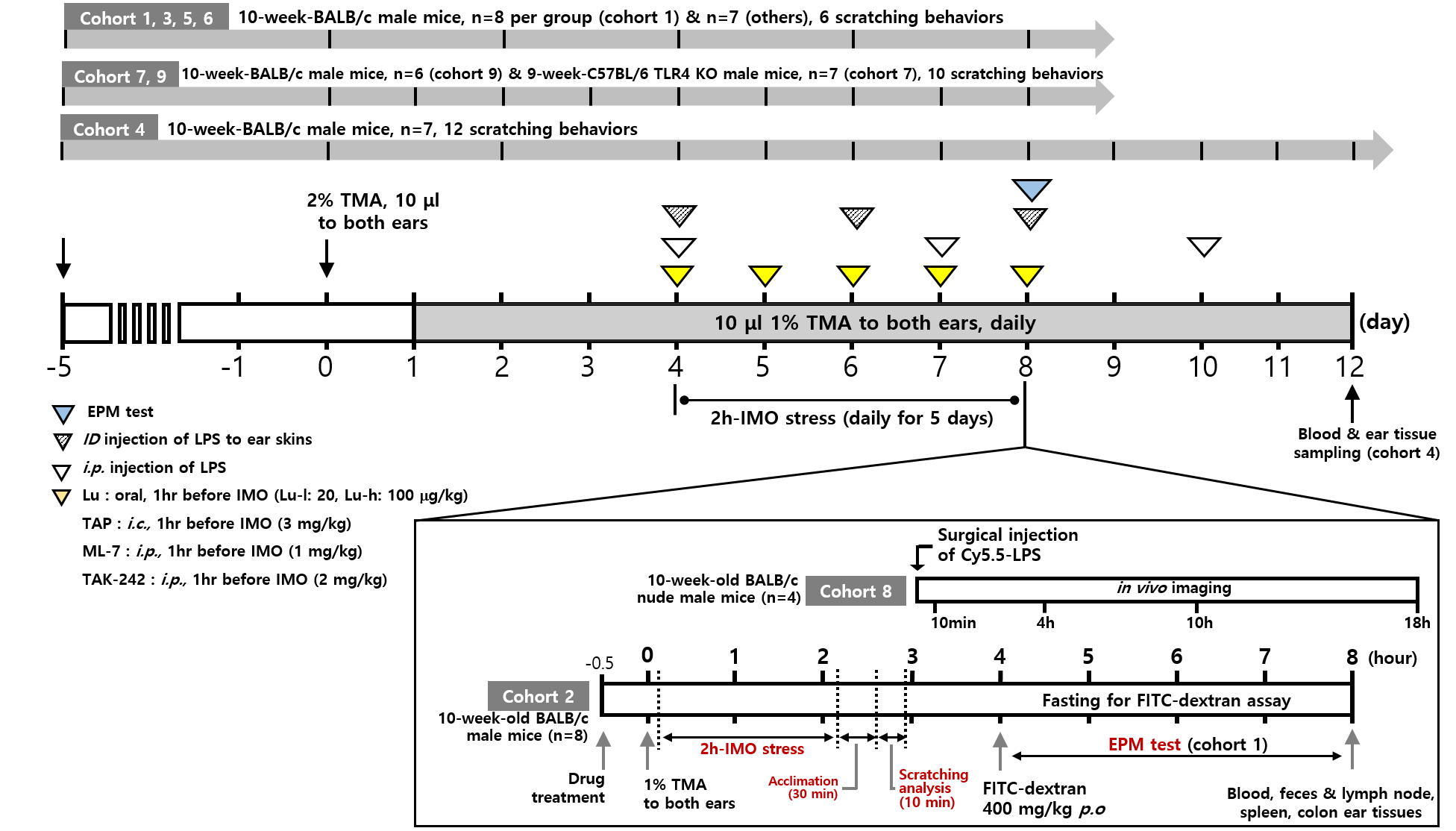


**Fig. S1** Cohort and experimental design. Nine- to ten−week−old naïve and nude BALB/c and C57BL/6 TLR4 KO male mice were used in this study, with a total of 9 cohorts participating. In cohorts 1, 3, 5, and 6, mice were examined for scratching behaviors at 6 time points, and mice in cohort 1 underwent an EPM test for 4 h after IMO stress on day 8. Short vertical lines in three right−bar arrows (gray) indicate the time points of analyzing scratching behaviors. EPM: elevated plus maze, TMA: trimellitic anhydride, IMO: immobilization, ID: intradermal, *i.p.*: intraperitoneal, *i.c.*: intracolonic, Lu: lubiprostone, TAP: 2,4,6−triaminopyrimidine, FITC: fluorescein isothiocyanate, Cy5.5: cyanine 5.5, LPS: lipopolysaccharide, KO: knockout, EPM: elevated plus maze.


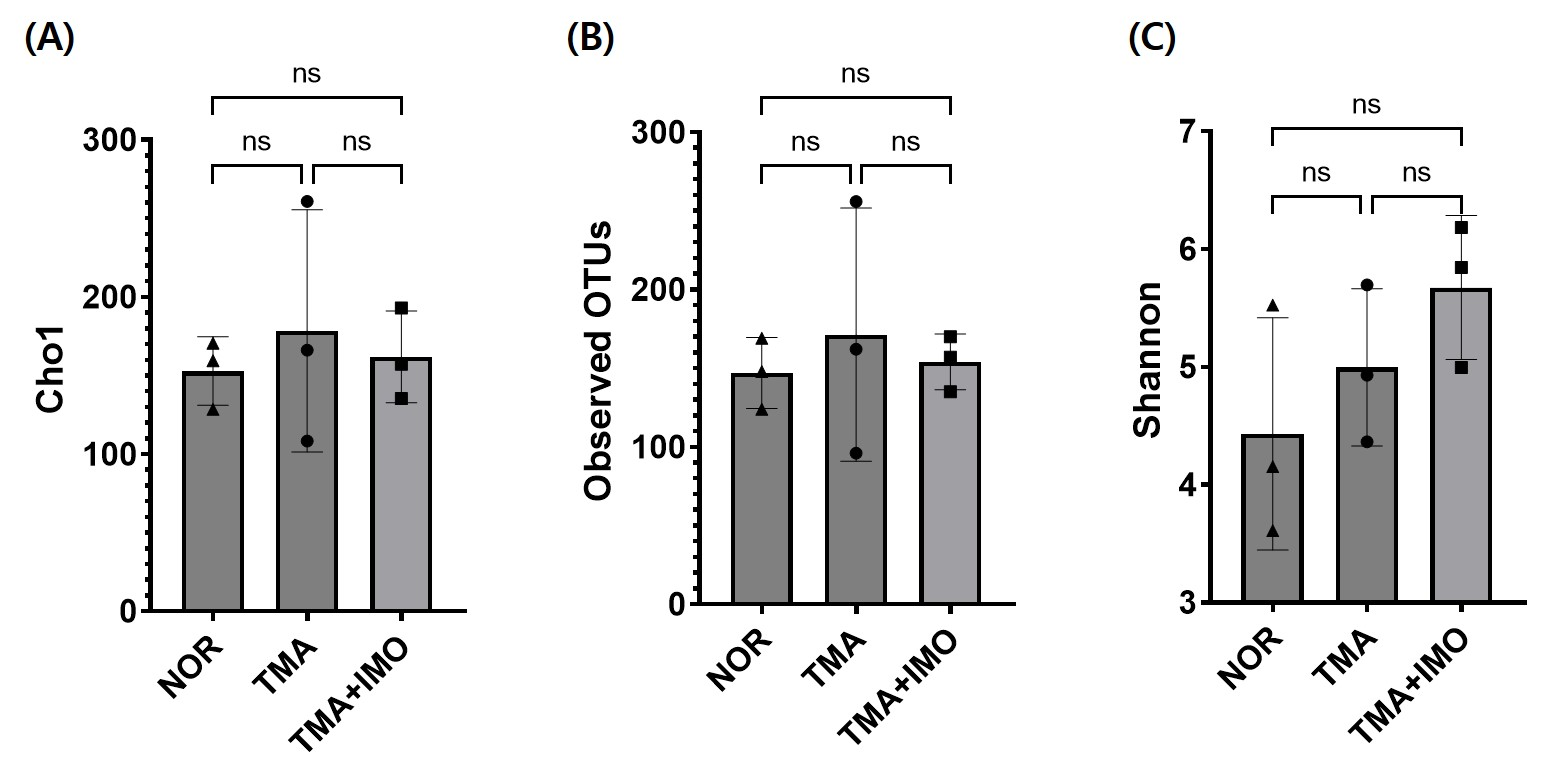

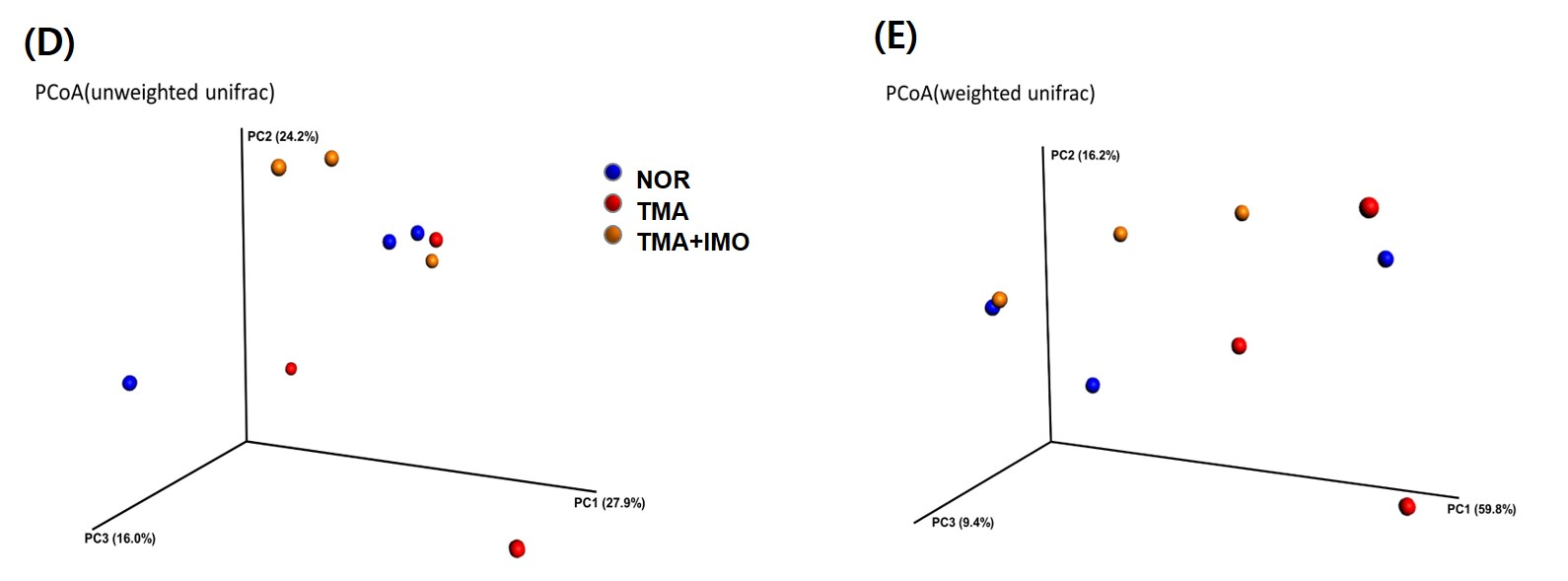


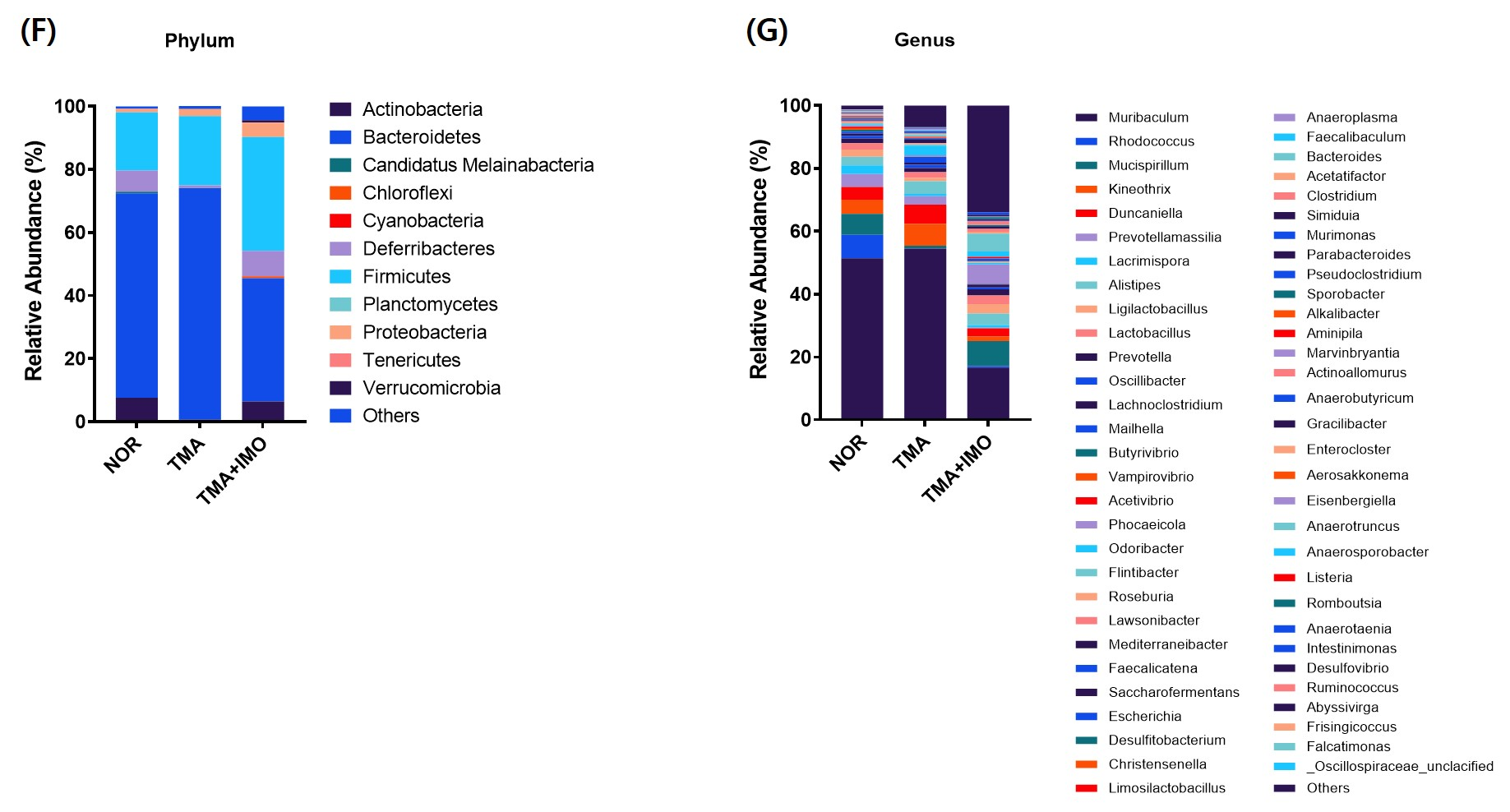


**Fig. S2** Comparison of α−diversity, PCoA, and bar plot analysis in the gut microbiota adhered to colon mucus layers of vehicle−treated NOR as well as TMA− and TMA+IMO−treated mice. There were no significant differences between groups in the comparison of the phylogenetic diversity index (A), Chao1 index (B), and Shannon index (C). Beta diversity was determined using weighted UniFrac phylogenetic distance matrixes visualized in principal coordinates analysis (PCoA) plots and tested for statistical significance by analysis of similarities (ANOSIM) with 999 permutations. PCoA plots based on unweighted (D) and weighted (E) distance matrixes calculated from OTU tables showing the separation among the gut microbiota adhered to the colon mucus layer were referred to as vehicle−treated normal (NOR, blue, n = 3), TMA−treated (TMA, red, n = 3), or TMA− and IMO stress−treated (TMA+IMO, yellow, n = 3). The relative abundance of the major phyla (F) and genera (G) was determined for NOR, TMA, and TMA+IMO mice. Others: Bacteria taxa with ≤ 0.2% abundance. TMA: trimellitic anhydride, IMO: immobilization.

**Supplementary References**

Chang CS, Liao YC, Huang CT, Lin CM, Cheung CH, Ruan JW, Yu WH, Tsai YT, Lin IJ, Huang CH, et al. 2021. Identification of a gut microbiota member that ameliorates DSS-induced colitis in intestinal barrier enhanced Dusp6-deficient mice. Cell Rep. 37: 110016.

Darwish N, Shao J, Schreier LL, Proszkowiec-Weglarz M. 2021. The choice of 16S ribosomal RNA primers affects microbiome analysis in chicken ceca. Sci Rep. 11: 11848.

Dicksved J, Schreiber O, Willing B, Petersson J, Rang S, Phillipson M, Holm L, Roos S. 2012. Lactobacillus reuteri maintains a functional mucosal barrier during DSS treatment despite mucus layer dysfunction. PLoS One. 7: e46399.

Lagkouvardos I, Lesker TR, Hitch TC, Gálvez EJ, Smit N, Neuhaus K, Wang J, Baines JF, Abt B, Stecher B, et al. 2019. Sequence and cultivation study of Muribaculaceae reveals novel species, host preference, and functional potential of this yet undescribed family. Microbiome. 7: 28.

Li H, Limenitakis JP, Fuhrer T, Geuking MB, Lawson MA, Wyss M, Brugiroux S, Keller I, Macpherson JA, Rupp S, et al. 2015. The outer mucus layer hosts a distinct intestinal microbial niche. Nat Commun. 6: 8292.

Litvak Y, Byndloss MX, Bäumler AJ. 2018. Colonocyte metabolism shapes the gut microbiota. Science. 362: eaat9076.

Schneider C, Döcke WD, Zollner TM, Röse L. 2009. Chronic mouse model of TMA-induced contact hypersensitivity. J Invest Dermatol. 129: 899–907.

Yeom M, Sur BJ, Park J, Cho SG, Lee B, Kim ST, Kim KS, Lee H, Hahm DH . 2015. Oral administration of Lactobacillus casei variety rhamnosus partially alleviates TMA-induced atopic dermatitis in mice through improving intestinal microbiota. J Appl Microbiol. 119: 560–570.

Méric G, Hitchings MD, Pascoe B, Sheppard SK. From *Escherich* to the *Escherichia coli* genome. *Lancet Infect Dis* 2016; **16**: 634–6.

Yuan T, Wang J, Chen L, Shan J, Di L. *Lactobacillus murinus* improved the bioavailability of orally administered glycyrrhizic acid in rats. *Front Microbiol* 2020; **11**: 597.

Ramasamy D, Lagier JC, Gorlas A, Raoult D, Fournier PE. Non contiguous-finished genome sequence and description of *Bacillus massiliosenegalensis* sp. nov. *Stand Genomic Sci* 2013; **8**: 264–78.

Rossi CC, da Silva Dias I, Muniz IM, Lilenbaum W, Giambiagi-deMarval M. The oral microbiota of domestic cats harbors a wide variety of *Staphylococcus* species with zoonotic potential. *Vet Microbiol* 2017; **201**: 136–40.

Xu X, Yu L, Xu G, Wang Q, Wei S, Tang X. *Bacillus yapensis* Sp. Nov., a novel piezotolerant bacterium isolated from deep-sea sediment of the yap trench, pacific ocean. *Antonie Leeuwenhoek* 2020*;* **113**: 389–96.

Pettersson B, de Silva SK, Uhlén M, Priest FG. *Bacillus siralis* sp. nov., a novel species from silage with a higher order structural attribute in the 16S rRNA genes. *Int J Syst Evol Microbiol* 2000; **50**: 2181–87.

Huang M, Aylin B, Bidhya S, Christiane M, Florian MWG, SS et al. *Bacillus firmus* I-1582 promotes plant growth and impairs infection and development of the cyst nematode *Heterodera schachtii* over two generations. *Sci Rep* 2021; **11**: 1.

Kun W, Wenxin L, Qichao T, Yichen G, Jinzhi H, Yu Z et al. Preliminary analysis of salivary microbiome and their potential roles in oral lichen planus. *Sci Rep* 2016; **6**: 1.

La Scola B, Mallet MN, Grimont PAD, Raoult D. *Bosea eneae* sp. nov., *Bosea massiliensis* sp. nov. and *Bosea vestrisii* sp. nov., isolated from hospital water supplies, and emendation of the genus BoSea (Das et al. 1996). *Int J Syst Evol Microbiol* 2003; **53**: 15–20.

Kassinen A, Krogius-Kurikka L, Mäkivuokko H, Rinttilä T, Paulin L, Corander J et al. The fecal microbiota of irritable bowel syndrome patients differs significantly from that of healthy subjects. *Gastroenterology* 2007; **133**: 24–33.

Gootz TD, Marra A. *Acinetobacter baumannii*: An emerging multidrug-resistant threat. *Expert Rev Anti Infect Ther* 2008; **6**: 309–25.

Takeuchi M, Hamana K, Hiraishi A. Proposal of the genus *Sphingomonas sensu* Stricto and three new genera, *Sphingobium*, *Novosphingobium* and Sphingopyxis, on the basis of phylogenetic and chemotaxonomic analyses. *Int J Syst Evol Microbiol* 2001*;* **51**: 1405–17.

Omer ZS, Tombolini R, Gerhardson B. Plant colonization by pink-pigmented facultative methylotrophic bacteria (PPFMs). *FEMS Microbiol Ecol* 2004; **47**: 319–26.

Fuerst JA, Hawkins JA, Holmes A, Sly LI, Moore CJ, Stackebrandt E. Porphyrobacter neustonensis gen. nov., sp. nov., an aerobic bacteriochlorophyll-synthesizing budding bacterium from fresh water. *Int J Syst Bacteriol* 1993; **43**: 125–34.

Gajdács M, Urbán E. The pathogenic role of *Actinomyces* spp. and related organisms in genitourinary infections: Discoveries in the new, modern diagnostic era. *Antibiotics (Basel)* 2020; **9**: 524.

Kristian S, Jakob TN, Steen VP, Bernhard P, Holger B, Frans AAM et al. Solution structure of the *Cutibacterium acnes*-specific protein RoxP and insights into its antioxidant activity. *Front Cell Infect Microbiol* 2022; **12**: 1–11.

Gumaelius L, Magnusson G, Pettersson B, Dalhammar G. *Comamonas denitrificans* Sp. Nov., an efficient denitrifying bacterium isolated from activated sludge. *Int J Syst Evol Microbiol* 2001; **51**: 999–1006.

Sabaté Brescó M, Harris LG, Thompson K, Stanic B, Morgenstern M, O’Mahony L et al. Pathogenic mechanisms and host interactions in *Staphylococcus epidermidis* device−related infection. *Front Microbiol* 2017; **8:** 1401.

Ding L, Yokota A. Proposals of *Curvibacter gracilis* gen. nov., sp. Nov. and *Herbaspirillum putei* sp. Nov. for bacterial strains isolated from well water and reclassification of [Pseudomonas] *huttiensis*, [*Pseudomonas*] *lanceolata*, [*Aquaspirillum*] *delicatum* and [*Aquaspirillum*] *autotrophicum* as *Herbaspirillum huttiense* comb. nov., *Curvibacter lansceolatus* comb. nov., *Curvibacter delicatus* comb. nov. and Herbaspirillum autotrophicum comb. Nov. *Int J Syst Evol Microbiol* 2004; **54**: 2223–30.

**Additional materials (Original PCR image):**

1. Full gel image for Fig. 3C


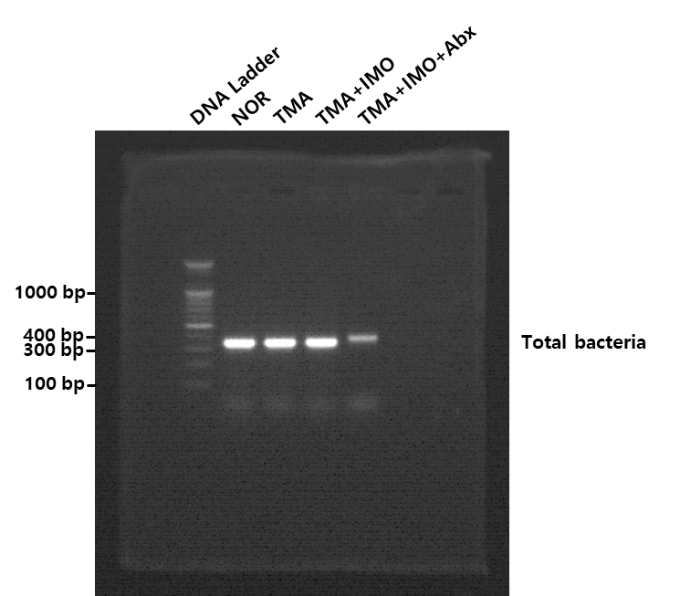


The picture is the original gel picture taken under ultraviolet (UV) light.
